# Emergence and Evolution of Hominidae-Specific Coding and Noncoding Genomic Sequences

**DOI:** 10.1093/gbe/evw132

**Published:** 2016-06-11

**Authors:** Morteza Mahmoudi Saber, Isaac Adeyemi Babarinde, Nilmini Hettiarachchi, Naruya Saitou

**Affiliations:** ^1^Department of Biological Sciences, Graduate School of Science, University of Tokyo; ^2^Division of Population Genetics, National Institute of Genetics, Mishima, Japan; ^3^Department of Genetics, School of Life Science, Graduate University for Advanced Studies (SOKENDAI), Mishima, Japan

**Keywords:** Hominidae, conserved noncoding sequences, lineage-specific genes, *DSCR4*, accelerated evolution

## Abstract

Family Hominidae, which includes humans and great apes, is recognized for unique complex social behavior and intellectual abilities. Despite the increasing genome data, however, the genomic origin of its phenotypic uniqueness has remained elusive. Clade-specific genes and highly conserved noncoding sequences (HCNSs) are among the high-potential evolutionary candidates involved in driving clade-specific characters and phenotypes. On this premise, we analyzed whole genome sequences along with gene orthology data retrieved from major DNA databases to find Hominidae-specific (HS) genes and HCNSs. We discovered that Down syndrome critical region 4 (*DSCR4*) is the only experimentally verified gene uniquely present in Hominidae. *DSCR4* has no structural homology to any known protein and was inferred to have emerged in several steps through LTR/ERV1, LTR/ERVL retrotransposition, and transversion. Using the genomic distance as neutral evolution threshold, we identified 1,658 HS HCNSs. Polymorphism coverage and derived allele frequency analysis of HS HCNSs showed that these HCNSs are under purifying selection, indicating that they may harbor important functions. They are overrepresented in promoters/untranslated regions, in close proximity of genes involved in sensory perception of sound and developmental process, and also showed a significantly lower nucleosome occupancy probability. Interestingly, many ancestral sequences of the HS HCNSs showed very high evolutionary rates. This suggests that new functions emerged through some kind of positive selection, and then purifying selection started to operate to keep these functions.

## Introduction

Family Hominidae which includes humans, chimpanzees, bonobos, gorillas and orangutans is one of the two living families of ape superfamily Hominoidea (see supplementary fig. S1, Supplementary Material online). Taxonomically this family belongs to the order Primates. All members of this family have large brains, well known for their complex social behavior and intellectual abilities. Facial expressions and intricate vocalization play a pivotal role in their behavior. Apart from humans, other species of this family have also shown signs of problem solving (Völter and Call 2012), the phenotype which have not been observed in other closely related species.

The disproportionately enlarged frontal cortex is believed to be mainly responsible for the uniqueness of human cognitive specialization. Several studies comparing human with nonprimates and non-Hominidae primates like baboon showed unique disproportionately enlarged frontal cortex in humans (McBride et al. 1999). However, by investigating frontal cortex of several primate species, including all extant hominoids using magnetic resonance imaging, Semendeferi et al. (2002) showed that human frontal cortex is not disproportionately larger than that of other great apes. Their findings clearly showed disproportionately enlarged frontal cortex to be a unique shared characteristic of the Hominidae family members and a distinctive feature compared with the rest of the species.

Despite the increasing genome data in the past decade, the genetic factors that contribute to the phenotypic uniqueness of Hominidae have remained elusive. Phenotype is the result of a collective network of genes along with other regulatory elements. Recent completion of the whole genome sequencing and gene annotation projects for a diverse variety of species, including the Hominidae family members and their closely related species has provided a strong foundation for comparative genomics analysis of lineage-specific characteristics.

So far, to identify the sequences underlying lineage-specific phenotypes within the Hominidae family, the majority of the studies have focused on detecting signatures of positive selection on humans using comparative genomics or genetic variation data produced by the International HapMap Project, Perlegen or 1000 Genomes Project. More than 20 genome-wide scans for positive selection have been performed on the human genome. Although the signals are not generally consistent, strongest signatures of positive selection were found to be on genes involved in host–pathogen interaction, immune response, reproduction (especially spermatogenesis), and sensory perception (Sabeti et al. 2006). Kosiol et al. (2008) studied signatures of positive selection on human–chimpanzee common ancestor as well as the common ancestor of Catarrhini, in which only 7 and 21 genes showed signs of positive selection, respectively. Positively selected genes in that study were also involved in immune response, reproduction and sensory perception. To date, positive selection on protein-coding genes has received the most attention as potential drivers of unique properties observed across the Hominidae family. However, there are other important aspects of the evolution of lineage-specific phenotypes which have so far been undervalued in Hominidae studies.

Clade-specific conserved noncoding sequences (CNSs) and clade-specific novel genes are high-potential evolutionary candidates, which may have been involved in driving clade-specific phenotypes. New genes have been revealed to be involved in the evolution of new molecular and cellular functions, developmental processes, sexual dimorphism and phenotypic diversity across species (Chen et al. 2013). Examining the evolutionary period of vertebrates provided evidence for accelerated new gene origination in the recent evolution of hominoids (Zhang et al. 2010). By analyzing expression profiles of human, chimpanzee and macaque, Blekhman et al. (2008) reported that taxonomically restricted genes may play a role in enabling organisms to adapt to changing environmental conditions. If the same scenario holds for clade-specific genes, it implies that the acquisition of new genes by the common ancestor of a particular clade may have played an important role in the development of adaptive novel clade-specific complex biochemical processes.

In addition to genes, CNSs have also been reported to determine lineage-specific characteristics. Eight percent of the human genome is speculated to be presently subject to negative selection and likely to be functional (Rands et al. 2014). CNSs are regions within the genome that are evolutionarily conserved despite not coding for proteins. To date, there have been numerous studies on the general features (Pennacchio et al. 2006; Mikkelsen et al. 2007; Babarinde and Saitou 2016) and evolutionary dynamics (Pennacchio et al. 2006; Faircloth et al. 2012) of CNSs, nearly all of which have proceeded to assign regulatory functions to these conserved genomic elements. CNSs have been reported to be linked to human disease (Visel et al. 2009). In stickleback, loss of a CNS containing a transcriptional enhancer regulating the pleiotropic *Pitx1* gene led to major phenotypic change (loss of pelvic spines) (Chan et al. 2010). In several studies in animals (Hiller et al. 2012; Takahashi and Saitou 2012; Babarinde and Saitou 2013) and plants (Hettiarachchi et al. 2014), CNSs are also proposed to be involved in lineage-specific phenotypes.

Lineage-specific duplication is yet another driving force of evolutionary changes across species. Studies of gene family evolution indicate that duplication events are enriched in primates and especially within ancestral branch leading to human and African great apes (Marques-Bonet et al. 2009). Although Hominidae ancestor do not show a strong burst in in duplication or deletion events as the common ancestor of African great apes, duplication and deletion might underlie some of the unique Hominidae lineage-specific traits. Analysis of duplication and deletion activities within Hominidae family have been conducted elsewhere (Marques-Bonet et al. 2009), and because the objective of this study is to discover Hominidae-specific (HS) unique genomic elements; we did not analyze duplication/deletion events in this article.

Here we explored the unique genomic elements underlying phenotypes restricted to the Hominidae family by identifying HS orphan genes and HS highly conserved noncoding sequences (HCNSs). We analyzed whole genome sequences along with gene expression and orthology data retrieved from databases to identify HS genes. We also analyzed Hominidae family members’ whole genomes along with those of gibbon, rhesus macaque and marmoset to discover HS HCNSs. Because of the short divergence time between Hominidae members and other closely related species, we used stringent thresholds for identifying HS orphan genes and HCNS to minimize type I error. We found that there is a low proportion of HS protein-coding gene to HS putative regulatory HCNSs, suggesting a likely stronger contribution of regulatory elements than novel genes in defining Hominidae-clade-specific phenotypes.

## Materials and Methods

### Retrieving Genome Sequence and Annotations

The human genome annotation was obtained from Gencode 19 (Encyclopedia of genes and gene variants) project (Harrow et al. 2012). For the rest of the species, genomic gene sets were retrieved from Ensembl release 75 FTP website. The repeat masked genome sequences of simians were retrieved from the Ensembl genome database. The genome nucleotide count used for identification of HS HCNSs in chimpanzee; gorilla and orangutan genomes are 2,902,322,413, 2,860,568,349 and 3,091,708,170, respectively. All the genomes are at least 5.6× coverage. The genomic coding coordinates were masked from genome sequences.

### Hominidae-Specific Genes

#### Homology Search for Detection of Homologous Genes

Phylostratigraphic analysis of gene age has been shown to be prone to erroneous gene age underestimation and substantially influenced by length of the encoded protein and its rate of evolution (Moyers and Zhang 2015). Young genes have been shown to be subject of weaker purifying selection (Cai and Petrov 2010) and encode shorter proteins (Wolf et al. 2009). Such characteristics of young genes have made accurate identification of HS genes challenging. In the study of Hominoid-specific de novo genes by Xie et al. (2012) six novel genes were found to be restricted to human, chimpanzee and orangutan, however, none of them could be identified as ape-specific protein coding gene using phylostratigraphic analysis of current DNA, protein and orthology databases (see supplementary table S1, Supplementary Material online). To minimize false positive results due to BLAST software limitations (Moyers and Zhang 2015) strict thresholds were used for identification of young genes restricted to Hominidae family.

**Fig. 1.**
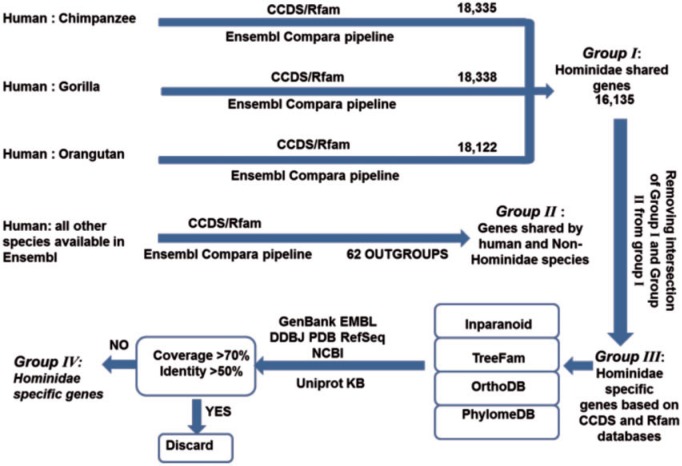
Hominidae-specific gene identification pipeline. Human was used as focal species and its genes were searched against the rest of Hominidae members’ genome to identify Hominidae shared genes, indicated by group I. Using the same strategy, pairwise orthologous genes were identified between human and outgroup species, indicated by group II. Intersection of Group I and Group II were omitted from Hominidae shared genes which gives rise to Hominidae-specific genes based on CCDS and Rfam databases. Group III genes were searched in orthology prediction databases (Inparanoid, Treefam, OrthoDB, PhylomeDB) along with DNA and protein databases (Genebank, EMBL, DDBJ, PDB, RefSeq, NCBI and Uniprot KB) and any of the gene queries with significant homology (coverage >70%, identity >50%) in non Hominidae members were discarded.

Experimentally verified human genes derived from Gencode project version 19 were selected as reference and searched against the other three Hominidae members’ genes using Ensembl Compara pipeline. Intersection of these three groups represents Hominidae shared genes. Using the same strategy, pairwise orthologous genes were identified between human and all non-Hominidae species available in Ensembl (see supplementary table S2, Supplementary Material online). The genes shared by Hominidae that are not present in outgroup species were searched in INPARANOID (Ostlund et al. 2010), TreeFam (Schreiber et al. 2014), PhylomeDB (Huerta-Cepas et al. 2011) and OrthoDB (Waterhouse et al. 2013) orthology prediction databases and the genes with orthologs in non-Hominidae members were discarded. NCBI MegaBLAST was recruited to search the remaining gene sequences in Genbank, EMBL, DDBJ, PDB and RefSeq database. NCBI BLASTP was also used to search HS protein-coding genes in UniprotKB database. Any of the gene queries with hits >70% coverage and 50% identity in non-Hominidae members was discarded. Summary of HS gene detection pipeline is depicted in figure 1.

#### Identifying the Evolutionary Origin of *HS Genomic Elements*

To identify evolutionary processes leading to the emergence of HS protein coding gene, its orthologous sequences in Hominidae closely related species, namely gibbon, rhesus macaque and marmoset, along with mouse were retrieved from pairwise whole genome lastZ alignments. The whole gene multiple sequence alignment of HS protein-coding gene was constructed using a combination of MISHIMA (Kryukov and Saitou 2010) and Mcoffee (Notredame et al. 2000). Neanderthal sequence homologous to HS protein coding genes were retrieved as short read alignments (Prufer et al. 2014) and were analyzed using SAMtools. At each position the nucleotide with highest average mapping quality and base quality score were chosen to construct HS protein coding gene in Neanderthal. Shotgun sequencing data of bonobo and baboon genome homologous to HS protein coding were, respectively, retrieved from NCBI (Prufer et al. 2012) and The Baylor College of Medicine Human Genome Sequencing Center (BCM-HGSC) and analyzed using Biopython.

To understand whether transposable elements have been involved in the evolution of HS gene, its exonic sequences were searched against transposable elements alignments and hidden Markov models of such elements using Repeatmasker and Dfam database (Wheeler et al. 2013). Analysis of the contribution of transposable elements in formation of human’s whole-genome protein coding exons (retrieved from Pfam database) was done using UCSC Galaxy.

#### Analysis of Selection

Codon-wise and nucleotide-wise analysis of selection using the method described by Haygood et al. (2007) was performed using HyPHY software (Pond et al. 2005). Analysis of selection in human populations was conducted on the 1,000 genomes data (Pybus et al. 2014). *EDAR* and *SLC24A5* genes were used as reference for measuring the significance of positive selection. Ectodysplasin A receptor coded by *EDAR* gene has been shown to be under positive selection in Asian populations (Bryk et al. 2008). Solute carrier family 24 member 5 coded by *SLC24A5* affects skin pigmentation and has undergone positive selection in European populations (Lamason et al. 2005). Analysis of selection on these two genes using three classes of population variation based tests, namely allele frequency spectrum (Tajima’s *D* test), linkage disequilibrium structure (EHH test) and population differentiation (XP-CLR test) (Chen et al. 2010) showed evidence of positive selection within these genes along with their flanking regions.

We measured signals of positive selection on HS protein coding gene along with its upstream and downstream flanking region using Tajima’s *D* test, EHH test and XP-CLR test. Signals of positive selection on *EDAR* and *SLC24A5* genes were used as positive control and were compared with that of HS protein-coding gene.

### Hominidae-Specific HCNSs

#### Setting the Percent Identity Threshold of Sequences under Purifying Selection and Neutrally Evolving Sequences

As the main objective of this study is to identify Hominidae-unique genomic elements evolved in Hominidae common ancestor ∼16–19 Ma (see supplementary fig. S1, Supplementary Material online), it is quite important to accurately differentiate between sequences that are under actual selective constraint and those that just did not have sufficient time to accumulate mutation. This fact is quite important due to the short evolutionary distance of 3.1 Ma between the emergence of the closest outgroup species used in this study which is gibbon and the emergence of the most distant member of Hominidae family, orangutan (see supplementary fig. S1, Supplementary Material online). To minimize the probability of false positives due to short divergence time, we set the threshold as 100% similarity in conservation and 100 bp in length for the identification of sequences under purifying selection.

For accurately determining the threshold for neutral evolution, we compared protein coding sequences’ synonymous site variation rate with that of noncoding genomic divergence rate between species; we considered the former as the depiction of neutral evolution rate in coding sequence and the latter as the neutral evolution rate in noncoding sequences. We retrieved the *d*_s_ values of one2one (with one to one correspondence in Ensembl biomart) orthologous protein coding genes for the human genes against gibbon, rhesus macaque and marmoset. To deal with the issue of unreasonably high *d*_s_ values we discarded 1% of outliers at the high end and constructed the distribution plot of *d*_s_ values. Mode of the plot was considered as the neutral evolution threshold in coding sequences. For setting the neutral evolution rate within noncoding sequences, after running pairwise noncoding BLAST search, we constructed the distribution plot of sequence divergence values. Mode of the plot was considered as the threshold of neutrally evolving sequences within noncoding sequences.

#### Homology Search for Noncoding Sequences

After masking coding sequences in each genome, we searched for sequences that are under selective constraint in the Hominidae family. To this end, we used human genome as reference query because of its high quality and availability of genome information, and used BLASTN 2.2.25+ (Altschul et al. 1997) to run whole genome pairwise homology search. The thresholds used were *E* value of 10 ^−5^ and database size of 3×10^9^. The *E* value cutoff of 10 ^−5^ with 100 bp size minimum length was proven to be efficient thresholds for identification of CNSs within primates (Babarinde and Saitou 2013). Nonchromosomal sequences (such as mitochondrial genome, unmapped DNA and variant DNA) were excluded. In the case of overlapping hits, only the longest hit was retained. Sequences under purifying selection within Hominidae family which had no homologs with conservation level above the neutral evolution threshold in outgroups were assigned as HS HCNS. In order to prevent erroneous identification of HS HCNSs as a result of repeatmasker software errors, UCSC netted chained files were used to map each HS HCNS in gibbon, rhesus macaque and marmoset unmasked genomes. HS HCNSs with conserved orthologous in interspersed repeats were also discarded.

#### Evolutionary Origin of HS HCNSs

To investigate the evolutionary origins of HS HCSNs, we mapped each of human HS HCNSs to gibbon and rhesus macaque genome sequences and aligned using ClustalW (Thompson et al. 1994). These alignments were concatenated and blocks with gaps were removed. Genetic distances were calculated using MEGA version 6 (Tamura et al. 2013).

#### Single Nucleotide Polymorphism and Derived Allele Frequency Analyses

We retrieved the final release of phase 3 variant set of 1000 Genomes project. For chimpanzee, gorilla and orangutan, genome variation data were retrieved from the Great Ape Genome Project (Prado-Martinez et al. 2013). Pyliftover and UCSC netted chain files were used to lift chimpanzee, gorilla and orangutan’s HCNS coordinates to the corresponding human hg18 coordinates. For each species we retrieved and combined Single Nucleotide Polymorphism (SNP) and insertion/deletion variation data and because the variations were mapped to human genome, we filtered out all variations with allele frequency of 1.0. For each of the three Great Apes, we generated random sequences with the same number and size as HS HCNSs in each species and investigated the coverage of variation in HCNSs and random sequences. For derived allele frequency analysis, we retrieved SNP frequency data of the Yoruba population of Nigeria, from the International HapMap project. The ancestral alleles of SNPs overlapping the HS HCNSs or random sequences were determined using pyliftover and chimpanzee sequence.

#### HS HCNS Flanking Region Conservation Level

We extracted HS HCNSs along with 2,000 bp upstream and downstream flanking sequences and aligned the sequences using ClustalW. For each alignment, we made sliding windows of 50 bp and step size of 20 bp starting from 30 bp inside the CNSs and calculated the percent identity in each window. We then calculated the average of the percent identity for each window.

#### Nucleosome Occupancy Probability

Kaplan et al. (2009) developed a probabilistic model of sequence nucleosome preferences. Considering dinucleotide signals along with favored and disfavored pentamer sequences in known nucleosome, this model produces a nucleosome occupancy score for each nucleotide of the subject sequence. Using version 3 of the nucleosome position prediction program, nucleosome occupancy probability for HS HCNSs were calculated considering 4,000 bp region at upstream and downstream starting from the center of the HS HCNSs. The average nucleosome occupancy probability was calculated for each nucleotide site of the total 8,000 bp along the length of sequences. The same procedure was carried out for random sequences of the same number and same size. Statistical significance was calculated using *t*-test for HCNS sites scores.

To confirm lower nucleosome occupancy of HS HCNSs, we retrieved genome binding/occupancy profiling data derived by high throughput sequencing and MNase-seq nucleosome positioning experiments from ENCODE/Stanford/BYU using UCSC (ftp://hgdownload.cse.ucsc.edu/golden Path/hg19/encodeDCC/wgEncodeSydhNsome/Gm12878Sig.bigWig, last accessed May 31, 2016). Average nucleosome occupancy score for HS HCNSs and flanking regions were calculated considering 4,000-bp region at upstream and downstream starting from the center of the HS HCNSs.

H3K9 methylation is the mark of heterochromatin regions. To further confirm the underrepresentation of HS HCNSs within heterochromatin regions, we retrieved H3K9me mapping data from ENCODE project and analyzed HS HCNS overlap with H3K9me histone mark compared with random sequences.

#### Genomic Distribution

We retrieved the annotations of the human genome from Gencode project and parsed each gene into regions, intersecting over alternative transcripts and splices, so that what are termed “UTR” and “intronic sites” are such sites with respect to all known transcripts and splices. We defined promoter region as the region within 1,000-bp upstream of a transcription start site. We then found HCNSs that are located on UTR, promoter, intronic and intergenic regions. We also calculated the fractions of UTRs, introns, promoters and intergenic sequences in the human whole-genome. Chi-squared test was used to analyze the significance of fraction differences.

#### Gene Ontology Analysis

We retrieved the coordinates of protein-coding genes from Gencode project. For HS HCNSs, we retrieved the list of genes found upstream and downstream of each HCNS. The gene that lies closest to a particular HCNS was considered as the likely target gene. If a HCNS was found inside a gene (including introns and UTR), the gene in which it resides was considered as the likely target gene. The likely target gene is with respect to the human reference genome. We checked the functional analysis of HS HCNSs using Panther 9.0 (Mi et al. 2010). P value corrected for multiple testing using Bonferroni correction was calculated. Unless otherwise stated, all scripts used for these analyses were written by one of us using Python or R and are available upon request.

#### Tissue Specificity of HS HCNSs

To investigate whether HS HCNSs do have unique properties in tissue-specific manner, we retrieved Dnase, chipseq and histone modification data for all tissues from Epigenome roadmap project (http://www.roadmapepigenomics.org/data/, last accessed May 31, 2016). The average score was calculated for each 400-bp window along the length of HS HCNS and flanking regions for the total of 18,500 bp. Standard error value for each window was calculated using SciPy (http://www.scipy.org/, last accessed May 31, 2016).

## Results

### Down Syndrome Critical Region of 4, HS Orphan Gene

By analyzing the DNA, protein and orthology databases, Down syndrome critical region of 4 (*DSCR4*) gene, on chromosome 21 discovered by Nakamura et al. (Nakamura et al. 1997) via EST mapping, was found to be the only annotated HS protein coding gene. *DSCR4* is an experimentally known gene, present in Ensembl, VEGA and consensus CDS protein set (CCDS) databases, and codes one known 117 amino-acid residue long polypeptide, one putative 127 amino-acid residue long polypeptide and a single 79 nonsense mediated decay transcript. However, although the 117 known amino acid long transcript is annotated in chimpanzee and orangutan, this transcript is missing in gorilla genome annotation. Close examination of the gorilla genome sequence revealed that the 117 amino acid long transcript could be constructed using gorilla–human orthologous sequences (see supplementary fig. S2a, Supplementary Material online); but it was not annotated due to limitations in the annotation algorithm. Analysis of Neanderthal and bonobo genome sequence homologous to human *DSCR4* sequence showed that the complete ORF could be successfully constructed in these two genomes indicating potential expression of *DSCR4* in all members of Hominidae whose genomes have been sequenced (see supplementary fig. S2a, Supplementary Material online). Although the expression data for placental tissue where *DSCR4* is mainly expressed is not available for great apes, expression analysis has detected *DSCR4* polyadenylated RNA in bonobo and chimpanzee testis as well as gorilla testis and heart (Brawand et al. 2011).

Proteins are generally composed of one or more functional domains. Combination of existing domains within a protein provides insights into the function of the protein. PFam database (Finn et al. 2010) contains high quality, manually curated protein domain entries named PFam-A along with automatically generated domain entries produced by Automatic Domain Decomposition Algorithm (ADDA) named Pfam-B. Searching *DSCR4* protein sequence within PFam showed no signs of homology to any known or predicted protein domain family. Examining uniprotKB database also revealed no homology to any existing protein sequence in any species other than Hominidae family. However, significant homology was found with yet uncharacterized proteins in all other members of Hominidae.

No experimental 3D structure analysis has been undertaken for *DSCR4* protein, nor were there any experimental structures with >90% sequence identity to *DSCR4* in protein 3D databases. However, the secondary structure of *DSCR4* based on Chou and Fasman algorithm (Chou and Fasman 1974) suggests the existence of potential α-Helices and β-Sheets (see supplementary fig. S3a, Supplementary Material online). Constructing the 3D structure by protein model portal (Arnold et al. 2009) and I-TASSER (Zhang 2008) also showed evidence for the existence of α helices and β sheets in the protein coded by *DSCR4* (see supplementary fig. S3b and *c*, Supplementary Material online).

**Fig. 2.**
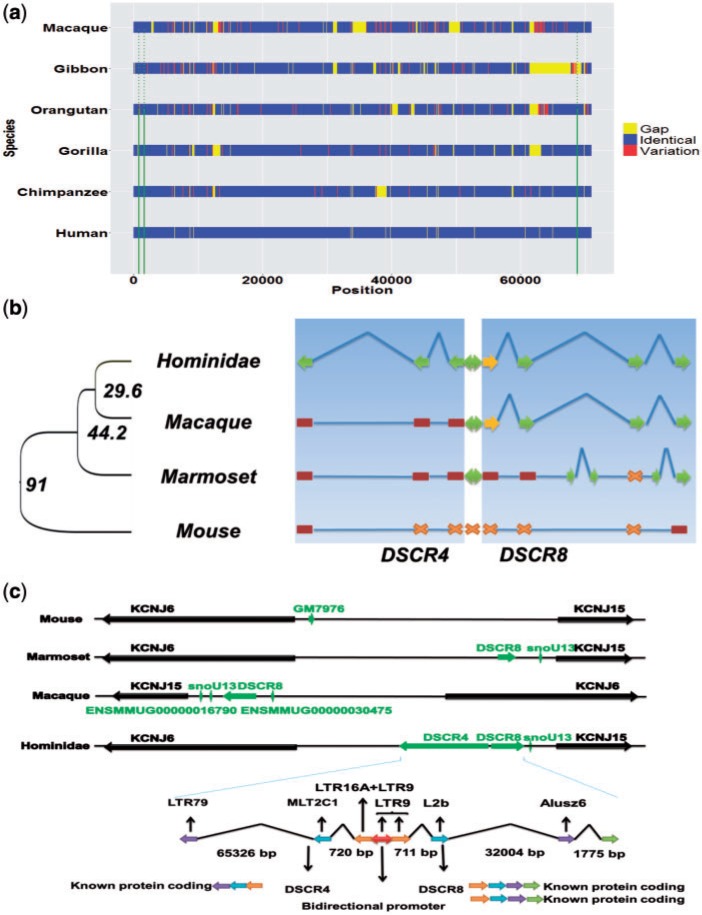
Evolutionary origin of *DSCR4* gene. (*a*) Multiple sequence alignment of *DSCR4* homologous sequences in Hominidae family members along with gibbon and rhesus macaque. Multiple sequence alignment for the sequences was undertaken using combination of Mishima, ClustalW and T-coffee. Identical and variant sites are defined based on Human genome reference sequence. (*b*) Schematic representation of the evolution of *DSCR4/8* genes and their shared promoter. Green arrows represent functional protein coding exons, yellow arrows represent exons coding only UTR, brown rectangles represent exons’ nonfunctional ancestral sequences and cross marks represent absence of homologous sequences for corresponding exon. (*c*) Evolution of genomic region located between *KCNJ6* and *KCNJ15* genes and contribution of transposable elements in formation of *DSCR4/8* genes along with their shared promoter.

Analyzing High coverage short-read data of gibbon genome (Carbone et al. 2014) revealed that *DSCR4* exon 3 coding sequence is partially missing in all sequenced gibbon individuals. This result indicates the possibility of lineage-specific deletion in gibbon genome sequence orthologous to human *DSCR4* gene. This observation is the sole reason for our shift to macaque genome as template for evolutionary analysis of the origin of *DSCR4* gene (fig. 2a).

*DSCR4* is separated by a 92-bp sequence from the *DSCR8* gene. The 92-bp separator sequence is part of a bidirectional promoter which initiates transcription from both of these genes. While *DSCR4* is limited to Hominidae family, *DSCR8* is present in Hominoidea, old world and new world monkeys. Multiple sequence alignment of DNA sequences corresponding to *DSCR4*/*DSCR8* gene and their shared promoter in Hominidae family along with closely related species and mouse suggests multi-step evolution of *DSCR4*.

Movement and accumulation of transposable elements (TE) have been a major force shaping the genes of almost all organisms (Feschotte and Pritham 2007). Investigating the role of TEs in evolution of human protein coding genes revealed 1.1% of all human protein coding exons to be at least partly derived from TEs (see supplementary fig. S4, Supplementary Material online). TEs also played a major role in the evolution of *DSCR4*/*DSCR8* genes. The first three exons of both *DSCR4* and *DSCR8* genes have been derived at least partly from transposons (fig. 2c).

By analyzing pairwise whole genome alignment data of Amniote lastZ (http://www.ensembl.org/info/genome/compara/analyses.html, last accessed May 31, 2016), evolution of *DSCR4* could be mainly classified in three evolutionary periods. Period (1) LTR79 retrotransposition that took place in the common ancestor of mammals >100 Ma. This transposition formed *DSCR4*’s exon 3 ancestral sequences. Period (2) During the evolution of common ancestor of primates at 29–45 Ma, three independent retrotranspositions by MLT2C1, LTR16A and LTR9 led to the formation of *DSCR4*’s exon 2, exon 1 along with *DSCR4/8* shared bidirectional promoter (see supplementary table S3, Supplementary Material online). Analysis of the core promoter region of *DSCR4/8* bidirectional promoter also reveals that the *DSCR4/8* bidirectional promoter region has retrotransposed and activated at this period (see supplementary fig. S5, Supplementary Material online). Period (3) The final ORF-enabling mutation was a GC transversion at *DSCR4* exon 3 that formed the stop codon TGA (fig. 2b, supplementary fig. S2b, Supplementary Material online). This transversion, which took place in the common ancestor of Hominidae 15–19 Ma, completed the formation of the *DSCR4* gene.

### Analysis of Selection

The 117 amino acid long, experimentally known transcript of *DSCR4* along with its orthologous sequences in other Hominidae species were used to examine signatures of selection. Codon-wise analysis of selection using Hyphy package showed no statistically significant signs of selection on any of the codons. Nucleotide-wise examination of selection also did not reveal any positively selected sites in promoter region. Population-based tests of selection (Tajima’s D, XP-EHH and XP-CLR) also showed no consistent sign of selection in any of European, Chinese or African populations (see supplementary fig. S6, Supplementary Material online). Analysis of the nonsynonymous and synonymous substitution rate also did not reveal evidence for strong purifying selection on this gene. These results are consistent with previous findings stating that young genes are subject to weaker purifying selection (Cai and Petrov 2010).

### Highly Conserved Noncoding Sequences

Gorilla diverged from the common ancestor of Homo and Pan Genera 8.8 Ma and later Homo and Pan diverged ∼6.9 Ma. The common ancestor of Hominidae diverged from Hylobatidae 18.8 Ma and 3.1 Myr later, orangutan, the most distant member of Hominidae family emerged (Hedges et al. 2015). Such short divergence times within family members and between Hominidae family and phylogenetically close species have made discerning HS functional noncoding sequences under purifying selection from neutrally evolving sequences a challenging objective.

The crucial parameter for identifying linage-specific CNSs in closely related species is the nucleotide identity threshold of the sequences evolving neutrally and sequences evolving under purifying selection. Due to short divergence times, false positive results are of high concern and thresholds are set in a way that takes special care of type I errors. As the majority of noncoding DNA sequences are assumed to be under neutral evolution (Kimura 1983; Saitou 2014), we considered the mode of the noncoding sequence alignment plot as the neutral evolution threshold. To verify the authenticity of this threshold, we also analyzed the neutral substitution rate in protein coding sequences. We constructed the synonymous substitution rate plot between human and three closest outgroup species, namely, gibbon, rhesus macaque and marmoset (see supplementary table S2 for scientific nomenclature, Supplementary Material online). In several studies, a number of synonymous sites in protein coding genes have been shown not to be strongly following neutral fashion. Some of these synonymous sites have been shown to be under weak selection constraint (Chamary et al. 2006) and may affect mRNA stability or splicing. On this premise, it is expected that protein coding’s *d*_s_-based neutral divergence plot to have similar distribution as the noncoding sequence identity-based plot but with a weak skew toward the conserved end. This pattern was indeed observed in pairwise comparison of human and all three outgroups (see supplementary figs. S7 and S8, Supplementary Material online) which suggests that our thresholds for neutrally evolving sequences are accurate. We filtered out all HS HCNS with orthologous sequences in any outgroups with divergence levels lower than neutral evolution threshold. Using this strategy we identified 1,658 HS HCNSs (HS HCNS coordinates, sequences and multiple alignment results are provided in supplementary material files, Supplementary Material online, in FASTA format). Length distributions of HS HCNSs are shown in supplementary figure S9 (see supplementary fig. S7, Supplementary Material online). Probability analysis using whole genome BLAST hits frequency data (see supplementary fig. S10, Supplementary Material online) showed that the frequency of sequences meeting all these conditions by chance is 3.88×10 ^−8^. As the total number of pairwise BLAST hits in each of reference genomes pairs are much <3.88×10^8^, it is extremely unlikely for HS HCNSs to be only cases of the outliers of neutral evolution.

### Functional Analysis of HS HCNSs

**Fig. 3.**
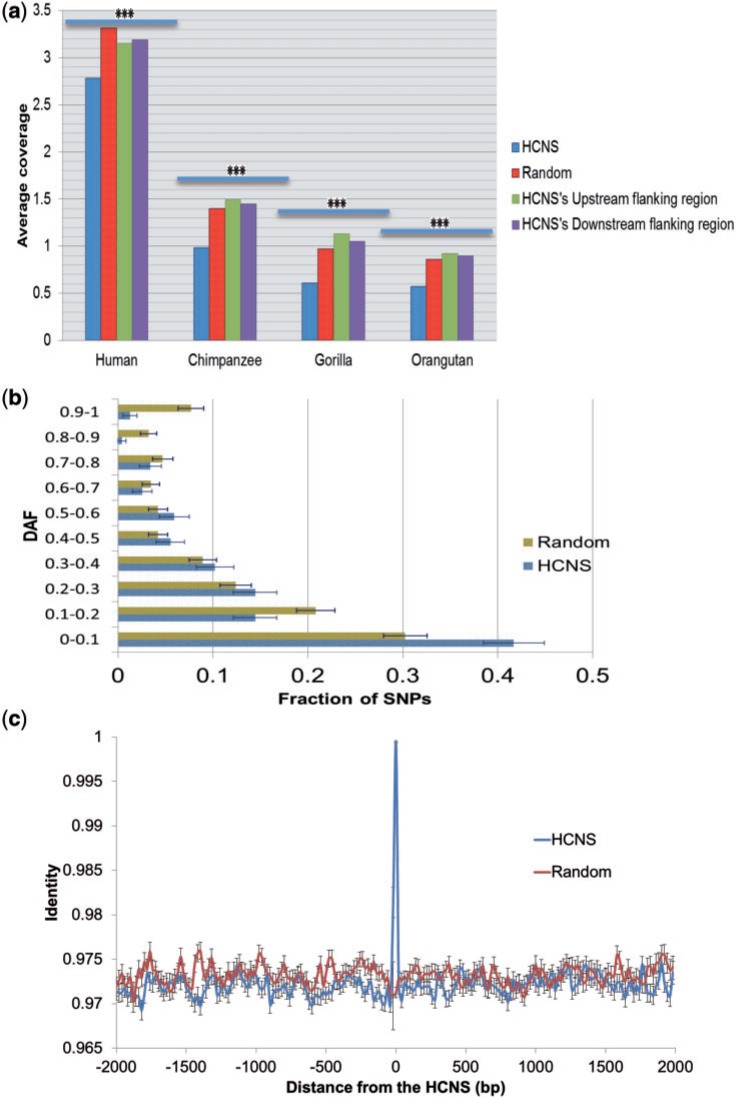
The polymorphism coverage and DAF analysis of HS HCNSs. (*a*) The average number of polymorphisms (SNP and INDEL) in 114 bp (average length of HS HCNSs) of HS HCNS along with HS HCNS flanking regions. Complete polymorphism data of 1,000 genome project along with polymorphisms with frequency less than one from great apes genome project were used. Polymorphisms are significantly underrepresented in HS HCNSs compared with random sequences (*t*-test *P* value < 10^−16^ for all members). (*b*) DAF distribution for Yoruba from Nigeria. Error bars were estimated using binominal distribution as $\text{σ=}\sqrt{\left(\text{pq}\right)/\text{N}}$, where *p* represents the fraction of polymorphisms in a particular bin, *q* represents (1−*p*), and N represented the total number of polymorphisms. (*c*) Conservation levels of HS HCNSs’ flanking regions. Point 0 is the average percent identity of 100 bp at the center of the HCNSs, whereas other points are the average of 50-bp windows moved at 20-bp steps starting from 30 pb inside the HCNSs. The standard error of the mean for each window is represented as error bars.

Genetic variation is a suitable indicator of selective constraint on a sequence. We investigated the frequency of SNPs, deletions and insertions overlaid on the HS HCNSs in human and great apes using 1,000 genome and great apes genome project data. The frequency of polymorphisms (SNP density per site: 2.4E-2, 8.6E-3, 5.3E-3 and 5.0E-3 for human, chimpanzee, gorilla and orangutan, respectively) in HS HCNSs are significantly lower than that of random sequences of the same number and same size (2.9E-2, 1.2E-2, 8.5E-3 and 7.5E-3) in all members of the Hominidae family (fig. 3a).

Derived allele frequency (DAF) analysis is another test of functionality of a sequence. Purifying selection is considered as the main evolutionary force to prevent CNSs from accumulating mutations. We found a higher proportion of HS HCNSs having lower derived alleles than random expectation. This suggests that HS HCNSs are under purifying selection (fig. 3b). At the level of DAF < 0.1, HS HCNS showed a significant excess of rare-derived polymorphisms compared with random expectations (Fisher test *P* value: 0.004) and by comparing all categories we noticed a significant shift in HS HCNS polymorphisms’ allele frequency toward rare allele frequencies (chi squared *P* = 0.001).

Are HS HCNSs located on local mutation cold spot regions in all the Hominidae family? To address this question, we checked the conservation level of the HS HCNS flanking regions. Figure 3c shows the pattern of conservation within HS HCNSs with up to 1,770 bp up- and down-stream flanking regions. For random sequences, unfiltered alignments of at least 2,000 bp long were used. The conservation plot indicates that only the HS HCNSs are highly conserved, indicating that they are under the strong constraints, relative to their flanking regions. We also investigated genetic variation frequency at upstream and downstream regions within the same length of each HS HCNS that do not overlap with known coding sequences. The genetic variations at HS HCNS up- and down-stream flanking regions are not significantly different from random noncoding sequences. However, their variation was significantly higher than that in HS HCNSs (fig. 3a). These results indicate that HS HCNSs are not located in mutation cold spots.

### Evolutionary Origin of HS HCNSs

How did HS HCNSs emerge? We need to compare outgroup species sequences of HS HCNSs to answer this question. Using whole genome mapping data, 32% (527) of HS HCSNs were mapped to gibbon and rhesus macaque genomes whereas the rest could not be mapped to these outgroup species genomes. We thus examined 527 multiple alignments of three sequences (HS HCNS, gibbon and rhesus macaque; see HS_HCNS_alignments.txt, Supplementary Material online). Length size distribution analysis revealed that average length difference of the mapped sequences in gibbon and rhesus macaque genomes from HS HCNSs is significantly higher than that of random sequences (see supplementary fig. S11, Supplementary Material online).

**Fig. 4.**
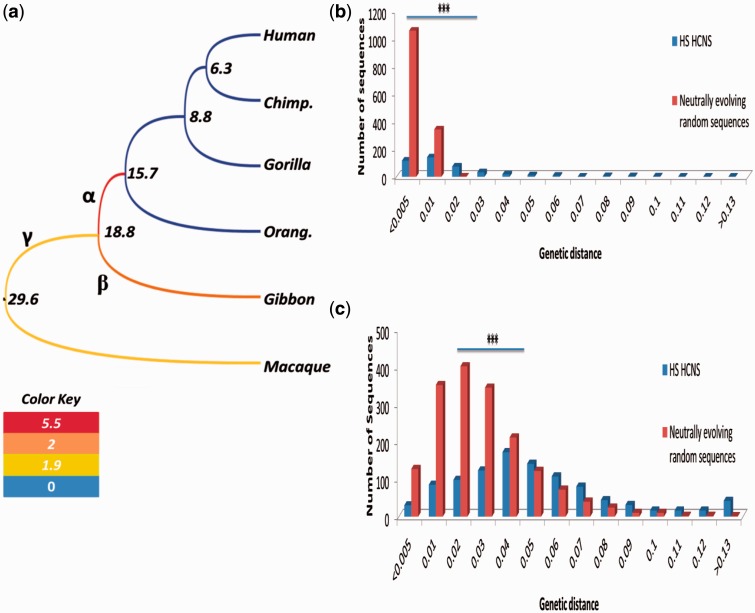
HS HCNS substitution rate across catarrhini phylogenetic tree. (*a*) catarrhini phylogenetic tree color-coded based on the substitution rate per million year in HS HCSNs orthologous sequences. Nucleotide substitution rates in rhesus macaque, gibbon and Hominoidea common ancestor in HS HCNS orthologous sequences are significantly higher than that of neutral evolutionary rate (represented as green in color key). Strongest accelerated mutation rate was observed in Hominidae common ancestor. (*b*) Comparison of genomic divergence in 32% of HS HCNS’s ancestral sequences in Hominidae common ancestor along with (*c*) 60% of HS HCNS’s orthologous and ancestral sequences in Hominidae common ancestor and gibbon with that of random sequences under pure neutral evolution reveals signature of accelerated evolution in HS HCNS orthologous and ancestral sequences.

We also estimated substitution rates (/site/year) at three branches (α, β, γ) of figure 4a for HS HCNSs orthologous and ancestral sequences using mapped gap-removed alignments. Divergence time estimates shown in supplementary fig. S1, Supplementary Material online, are used for rate estimations. We are particularly focused on branch on branch α of the phylogenetic tree shown in figure 4a, because this branch corresponds to the common ancestor of Hominidae after divergence of the common ancestor of Hominidae and Hylobatidae (gibbons). The mean rate of nucleotide substitution at branch α was 5.5×$10^{-9}$ (fig. 4a), which is 5 times higher than that (1.1×$10^{-9}$) of the neutrally evolving genomic regions. Interestingly, the substitution rates for branches β and γ (2×$10^{-9}$ and 1.9×$10^{-9}$, respectively) were also higher than the neutral rate (fig. 4a).

A very high mean substitution rate for branch α of figure 4a suggests the existence of positive selection at this branch followed by purifying selection in the later Hominidae lineages. We therefore examined the distribution of substitution numbers at branch α for those 527 HS HCNSs, as shown in blue bars of figure 4b. Red bars of figure 4b are corresponding to distribution of 1,658 randomly chosen sequences, which are considered to be under pure neutral evolution. Distribution patterns of blue and red bars are clearly different, and a total of 97 (18% of 527) HS HCNSs showed the rates >0.02, the largest branch length value observed for some purely neutral genomic regions. This suggests that at least 18% of HS HCNSs experienced some kind of positive selection which enhanced their substitution rates.

We found that 527 HS HCNSs were orthologous both to gibbon and rhesus macaque sequences. However, there were 1,001 HS HCNSs whose orthologs were found only in gibbons. In this case, without rhesus macaque, we cannot distinguish branches α and β. Yet, if the average of these two branches again showed elevated substitution rates, our finding based on only 527 HS HCNSs can be strengthened. In fact, the mean substitution rate for branches α and β combined for 1,001 HS HCNSs was 2.3×$10^{-9}$ (fig. 4c). If we subtract the contribution of branch β from this rate, we obtain the new substitution rate estimate (3.9×$10^{-9})$ for branch α. This value is slightly lower than that 5.5×$10^{-9}$ for 527 HS HCNSs, but still >3 times higher than the neutral rate. This confirms an elevated nucleotide substitution rate at branch α. Branch length distribution of those 1,001 HS HCNSs is shown as blue bars in figure 4c with random regions shown in red bar. Branch lengths for HS HCNSs are clearly shifted to larger ones compared with purely neutral ones.

These results indicate that insertions and deletions along with accelerated evolution in the common ancestor of Hominidae are the main evolutionary changes leading to the formation of HS HCNSs. We examined neighboring genes of these HS HCNSs with very high substitution rate for branch α of figure 4a. Supplementary table S4 of supplementary Material online lists these genes. NADPH oxidase (NOX) 3 is a member of the NOX/dual domain oxidase family with 50-fold overexpression in inner ear. *Nox3* is indispensable gene in formation of otoconia within inner ear (Paffenholz et al. 2004). *Sall3* is a member of splat gene family. Mutations in members of this family have been associated with several congenital disorders (Sweetman and Munsterberg 2006). *ABCD4* is a member of the superfamily of ATP-binding cassette (ABC) transporters involved in peroxisome biogenesis and adrenoleukodystrophy (ALD) disorder (Matsukawa 2011). The cocaine- and amphetamine-regulated transcript peptide (*CARTPT*) is involved in reward and feeding behavior and function as a psychostimulant (Lohoff et al. 2008). *TPRXL* and *MAGEA1* which are involved in embryonic development are among the likely target genes of highly conserved HS HCNSs.

### Genomic Distribution of HS HCNSs

We investigated the genomic location of each HCNS to examine whether there is any general trend in the distribution of HS HCNSs. HS HCNSs were categorized into four classes: intergenic, intronic, UTR and promoteric. Distribution of HS HCNS within these categories is shown in table 1. Their distribution significantly differs between HS HCNSs and rest of the genome (*P* value = 2.2E-16, chi-squared test). The fraction of HS HCNSs residing in introns, UTR and promoter regions of the human genome are significantly higher than those of the whole genome. The fractional increments are especially prominent in UTR (>3 times higher than the whole genome fraction) and promoter regions (almost 2 times higher than the whole genome). The increased proportions of HS HCNSs within UTR and promoter regions are consistent with previous findings of the genomic distribution of CNSs in primates (Takahashi and Saitou 2012; Babarinde and Saitou 2013) who reported the notably increased fraction of CNSs in UTR and promoter regions.

**Table 1:** Fractions (%) of Genomic Categories in HS HCNSs and the Human Genome

	HS HCNS^a^	Human Genome
Intergenic	59.5 (1,102)	74.4
Intronic	38.0 (703)	24.6
Promoter	1.1 (21)	0.6
UTR	1.4 (26)	0.4

^a^Absolute numbers are given in parentheses.

### Prediction of Nucleosome Positioning

**Fig. 5.**
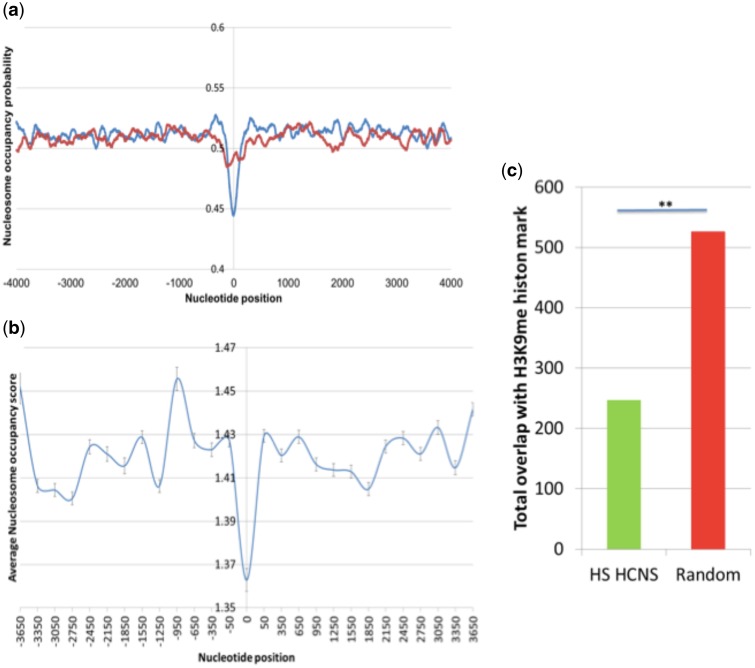
Nucleosome occupancy probability for HS HCNSs including flanking regions. (*a*) Zeroth nucleotide position represents the center of HCNSs and also the center of the random samples. Blue and red graphs show nucleosome occupancy probabilities of the HS HCNSs and random samples, respectively. (*b*) HS HCNS average nucleosome occupancy score derived from genome occupancy profiling generated by ENCODE/Stanford/BYU. HS HCNSs do have significantly lower nucleosome occupancy compared with flanking regions. (*c*) HS HCNS overlap with H3K9me histone mark compared with random sequences. H3K9 methylation is the mark of heterochromatin. HS HCNSs are significantly underrepresented in H3K9me marked regions defined by ENCODE project.

Nucleosome positioning with respect to DNA plays a crucial role in transcription regulation. Packing DNA in nucleosomes can limit the accessibility of the sequences and low nucleosome occupancy is considered as an important feature of transcription factor binding site (TFBS) (Miele et al. 2008; Schones et al. 2008). We computed the nucleosome position probability of HS HCNSs and their flanking regions using the nucleosome prediction probability algorithm developed by Kaplan et al. (2009), 4,000 bp region from the center of each HS HCNS at both upstream and downstream. A clear drop in nucleosome occupancy was observed directly overlapping with the center of HCNSs indicating the possibility of nucleosome depletion within the HS HCNS regions (fig. 5a). The nucleosome occupancy probability within HCNS regions was significantly lower than the random expectations (*P* value = 4.149E-40, *t*-test). This result was further confirmed using experimental genome occupancy profiling data derived by high throughput sequencing and MNase-seq nucleosome positioning experiments (fig. 5b). Analysis of H3K9me heterochromatin mark also revealed significant underrepresentation of HS HCNSs within H3K9me-marked heterochromatin regions (fig. 5c). Babarinde and Saitou (2013) discussed the possibility of their low-GC mammalian CNSs as also found for HS HCNSs (see supplementary fig. S12, Supplementary Material online) to nucleosome occupancy, and Kenigsberg and Tanay (2013) found a similar nucleosome positioning pattern in Drosophila CNSs.

### Gene Ontology Analysis

We considered the closest genes to HS HCNSs as the likely target gene on the premise that regulatory elements reside in close proximity with the gene they regulate, and examined the enrichment of biological process of HS HCNSs using PANTHER. Ninety-seven percent of HS HCNSs are located within 1 Mb of their nearby protein coding gene, the range in which most of gene regulatory elements are located (see supplementary fig. S13, Supplementary Material online). This observation is significantly different from the random expectation (*P* value: 1e-05, empirical chi-squared test; for null distribution, see supplementary fig. S14, Supplementary Material online). However, growing number of human genetic conditions are being found resulting from mutations in regulatory elements located >1 Mb away from the gene they regulate (Ghiasvand et al. 2011; Symmons and Spitz 2013). As a result, the possibility of the remaining 3% of HS HCNSs being regulatory elements of their nearby genes could not be ruled out.

Table 2 shows the top categories in which HS HCNSs are enriched. The gene enrichment analysis for the HS HCNS target genes indicate that sensory perception of sound has the highest fold enrichment among significantly overrepresented biological function categories. *PCDH15* and *cdh19* are two auditory critical genes located in close proximity of HS HCNSs. Protocadherin 15 (*PCDH15*) mutations in which causes inherited deafness called usher1F syndrome (Sotomayor et al. 2012) is the likely target gene of two HS HCSNs found in this study. *PCDH15* plays a crucial role in mechanotransduction that is important for sound characterization in the inner ear. Cadherin 19 (*cdh19*) is another likely target gene of two HS HCSNs, down-regulation of which has been linked to the development of cholesteatoma, an expanding destructive epithelial lesion within the middle ear (Klenke et al. 2012). Analysis of the gene ontology of random sequences did not show any enriched category of biological functions.

**Table 2:** Gene Ontology of HS HCNS-Associated Genes

Biological Process	Fold Enrichment
Sensory perception of sound	3.40
Cell–cell adhesion	1.76
Mesoderm development	1.68
Cell adhesion	1.68
Biological adhesion	1.68
System process	1.58
Neurological system process	1.57
System development	1.53
Multicellular organismal process	1.48
Single-multicellular organism process	1.48
Developmental process	1.42
Cell communication	1.28
Cellular process	1.25

Consistent with previous analysis of CNSs (Takahashi and Saitou 2012; Babarinde and Saitou 2013), genes involved in developmental process are also mainly enriched as likely target genes of HS HCNSs (table 2). *Fox* and *Sox* gene families play critical roles in the process of development. The *FOX* gene family genes are involved in developmental processes, organogenesis and speech acquisition (Hannenhalli and Kaestner 2009). Several members of this family, including *FOXD4L2*, *FOXD4L5*, *FOXD4L4*, *FOXK1*, *FOXE1*, *FOXR2*, *FOXI2*, *FOXG1* and *FOXN3* are in close proximity with HS HCNSs. Among the other likely target genes of HS HCNSs are *SOX1*, *SOX5* and *SOX11*. These genes are members of the *SOX* gene family that is also involved in regulating several crucial aspects of development (Prior and Walter 1996).

Brawand et al. (2011) analyzed the evolution of gene expression in mammalian organs and identified numerous genes with expression switch on the branch connecting great apes and macaque. These reported genes are the target of 158 HS HCNSs (see supplementary table S5, Supplementary Material online) with enrichment of the expression in cerebellum that is associated with language processing, learning, addiction and motor functions (Strick et al. 2009). This result is significantly different from random expectation (*P* value: 0.00767, empirical chi-squared test) (see supplementary fig. S14 for null distribution, Supplementary Material online). These results indicate the possibility of the evolution of HS HCNSs as the regulatory elements responsible for gene expression switches contributing to specific organ biology of Hominidae family.

Analysis of tissue specificity of HS HCNSs also revealed that HS HCNSs have, respectively, intensified average chromatin immunoprecipitation signal and H3K4me3 epigenetic mark within fetal brain and placenta compared with flanking regions (see supplementary fig. S15, Supplementary Material online). H3K4me3 is associated with active promoter regions. These data are in line with overrepresentation of HS HCNSs in promoter regions and enrichment of developmental process in Gene ontology analysis of likely target genes of HS HCNSs. These results give evidence for the likely role of HS HCNSs as regulatory elements mainly involved in development which have been suggested to play key roles in phenotypic diversity across species (Carroll 2000).

Comparing properties of HS HCNSs with human genome regions under accelerated evolution (HARs) identified by Pollard et al. (2006) and CNSs under accelerated evolution in human (HACNs) identified by Prabhakar et al. (2006) revealed no significant overlap (see supplementary table S6, Supplementary Material online). These results were expected due to significant difference not only in the direction of evolutionary changes but also in the time intervals in which HARs, HACNs and HS HCNSs were under action of evolutionary forces, indicating age-dependent properties of CNSs, as also suggested by Babarinde and Saitou (2013).

Analysis of lincRNA from Ensembl, enhancer sequences from Fantom project (http://fantom.gsc.riken.jp/data/, last accessed May 31, 2016) and GWAS-tagged SNPs from NHGRI-EBI (http://www.ebi.ac.uk/gwas/, last accessed May 31, 2016) also showed neither significant overrepresentation of HS HCNSs in lincRNA or enhancer sequences nor enrichment of GWAS-tagged SNPs suggesting that the mode of action of the majority of these elements under strong purifying selection are yet to be fully understood

## Discussion

Unraveling the molecular mechanisms underlying unique cognitive specialization shared by humans and great apes such as language learning and problem solving ability has been of particular interest to researchers from a broad range of scientific fields and so far, several comparative genomic studies have been conducted to explore the genomic sequences underlying human-specific phenotypes (Pollard et al. 2006; Prabhakar et al. 2006; Sumiyama and Saitou 2011). However, due to unavailability of high throughput sequencing technology and whole genome data for apes until the first decade of new millennium, molecular evolutionary genetics has not progressed as much in deciphering underlying genomic components of HS unique phenotypes. Emergence of novel genes has been linked to appearance of novel developmental and behavioral phenotypes in several species. Examples include dry-nosed primate-specific insulin-like 4 (Arroyo et al. 2012), Arabidopsis-specific *CYP84A4* (Weng et al. 2012) and Drosophila-specific *Xcbp1* genes (Chen et al. 2012) which, respectively, affect fetal development, pollen development and foraging behavior. Although emergence of lineage-specific genes have been shown to be a major contributor to adaptive evolutionary innovation, there are still gaps in evolutionary genomics in explaining lineage-specific characteristics and phenotypes which could not be answered by mere presence or absence of a particular set of genes. Within several kingdoms of species, lineage-specific CNSs have been suggested to be involved in spatiotemporal regulation of gene expression (Janes et al. 2011; Babarinde and Saitou 2013; Hettiarachchi et al. 2014). Although the specific functions of these conserved elements are mainly unknown, functional analyses have shown CNSs to be under purifying selection and enriched in close proximity of genes involved in developmental process in mammals and amniotes (Janes et al. 2011; Takahashi and Saitou 2012; Babarinde and Saitou 2013). As phenotypic evolution has been suggested to be primarily mediated by genes involved in developmental process (Nei 2007), CNSs could be considered as a high-potential candidate for filling the knowledge gap in elucidating the molecular basis of phenotypic diversity across lineages.

In this study, we identified one HS protein-coding gene and 1,658 CNSs originated in the common ancestor of Hominidae. As comprehensive analysis of gene expression has not yet been uniformly accomplished for Hominoids and monkeys, projection of human’s experimentally verified genes in great apes and monkeys were used as the sets of existing genes. We defined HS HCNSs as homologous regions with at least 100 bp length and conservation level of 100% within Hominidae members with no orthologous sequence with conservation level above neutral evolution threshold in non-Hominidae simians. Although it is possible that some putative genes with undetected expression or CNSs with less degree of conservation are functional, we assume that our conservative approach for HS novel gene and HS HCNS identification screens only genomic elements that are functionally important to Hominidae.

Down syndrome critical region has long been known to include genes involved in higher brain functions. This region has also been proposed to be responsible for the mental retardation phenotype observed in Down syndrome which is characterized by verbal short-term memory, spatial learning and deficits in speech and language (Olson et al. 2007). The critical importance of this region is consistent with our discovery that the only experimentally known HS protein coding gene is placed in the DSCR region. Although the fact that this protein is mainly derived from transposable elements with no homology to any family of proteins raises doubts about the functionality of this protein, there are numerous evidences at RNA and protein level, indicating the functionality of this gene. These evidences are: (1) higher absolute expression values compared with flanking conserved genes (see supplementary fig. 16, Supplementary Material online), (2) tissue-specific expression (Uhlén et al. 2015), (3) epigenetic marks for active regulatory region (see supplementary fig. 17, Supplementary Material online), (4) being a binding site of several transcription factors (see supplementary fig. 17, Supplementary Material online), (5) the likely existence of secondary structures in *DSCR4*-coded protein (see supplementary fig. S2, Supplementary Material online) and (6) acting as a fetal epigenetic marker for detection of Down syndrome (Du et al. 2011). These evidences indicate active regulation and expression of *DSCR4*, which in turn suggests this gene to be a functional element in humans. Further functional analysis of *DSCR4* might lead to better understanding of the genomic pathways involved in development of higher brain functions shared by Hominidae members and affected in Down syndrome.

Spatiotemporal regulation of gene expression has long been reported to be important in phenotypic diversity (Carroll 2000). The conservation level, coverage of polymorphism as well as DAF analysis supports that the potential HS regulatory elements identified as HS HCNSs are under functional constraint and may be involved in regulatory functions restricted to members of Hominidae family. Nucleosome positioning analysis showed low nucleosome occupancy probability in HS HCNSs implying that these elements have lower probability to form nucleosomes. The finding by Bai and Morozov (2010), stating that regulatory sequences are more nucleosome-depleted, gives additional support to the hypothesis that HS HCNSs is functional and involved in transcriptional regulation of their target genes.

According to our finding, insertions and deletions along with accelerated substitution rate in the Hominidae common ancestor are the main driving force for the evolution of HS HCNSs. Lineage-specific accelerated evolution in noncoding sequences have been proposed to be involved in evolution of species, potentially through lineage-specific changes in gene regulation (Bird et al. 2007). Evidence of prominent accelerated evolution on mappable HS HCNS ancestral sequences followed by strong purifying selection found in our study suggests that HS HCNSs have played key role in the emergence of Hominidae as a unique lineage among primates.

Gene ontology analysis carried out for HS HCNSs suggests HS HCNSs to be located close to genes mainly involved in developmental processes. Previous genome analyses of animals and plants have also demonstrated CNSs to be located near genes involved in developmental process. These findings agree with the idea that differences in the cis-regulatory elements involved in developmental process have a central role in intraspecific variation and phenotypic diversity across species (Carroll 2000) and gives further evidence for the contribution of HS HCNSs to the characteristics uniquely shared by Hominidae members. One interesting feature to note is the highest fold enrichment of likely target genes of HS HCNSs for the sensory perception of sound. Unlike the enrichment for developmental process which is shared between conserved elements within several lineages, sound sensory perception is uniquely overrepresented in HS HCNSs target genes. Sensory perception of sound is defined as the series of events required for an organism to receive an auditory stimulus, convert it to a molecular signal, and recognize and characterize the signal (Mi et al. 2013). Considering the unique sophisticated linguistic abilities observed within Hominidae (Patterson and Linden 1981; Savage-Rumbaugh et al. 1985; Miles and Miles 1990), one plausible reason to explain this observation is that HS HCNSs might be involved in development of unique sound sensory systems required for recognition and characterization of intricate communicative sounds used by humans and great apes.

Comparing genome-wide analyses of primate-specific genes (measured as transcriptional unit) and primate-specific gene regulatory elements (measured as primate-specific HCNSs) shows that the ratio of lineage-specific protein coding genes to lineage-specific highly conserved regulatory elements is only 0.007 (59/8,198) (Tay et al. 2009; Takahashi and Saitou 2012). The HS protein coding gene to HS HCNS ratio, 0.0006 (1/1,658) found in this study, is more than 1/10 lower than the already low primate-specific gene to HCNS ratio. These results are consistent with the notion that the morphological diversity is mainly accounted for by differences in regulatory elements (Carroll 2000), suggesting regulation alteration of existing protein-coding genes might have played a more significant role in Hominidae evolution than emergence of novel genes.

In this study, we identified one HS protein coding gene and 1,658 potential regulatory HCNSs originated in the common ancestor of Hominidae clade members 15–19 Ma. Although young, tissue-specific genes are of high medical relevance, functional characterizations of human genes have been biased against these genes (Hao et al. 2010). The Hominoide-specific protein coding gene *DSCR8* and HS protein coding gene, *DSCR4*, are examples of such bias which despite being placed on medically important region, DSCR of chromosome 21, their structure and function are not studied yet. In this study, HS HCNSs are shown to be under accelerated evolution in the Hominidae common ancestor, overrepresented in promoters, untranslated regions and in close proximity of genes involved in sensory perception of sound and developmental process. They also showed a significantly lower nucleosome occupancy probability. This study provides candidates of genes and regulatory elements which are expected to hold the key to the understanding of the phenotypic uniqueness shared by human and great apes, via mechanisms majority of which are yet to be fully understood. Experimental verification of these elements is expected to shed light on the lineage specificity of Hominidae.

## Supplementary Material

Supplementary material files, figures S1–S17 and tables S1–S6 are available at *Genome Biology and Evolution* online (http://www.gbe.oxfordjournals.org/).

Supplementary Data
