# Supplementary material for: Emergence and Evolution of Hominidae-Specific Coding and Noncoding Genomic Sequences
Source: Genome Biol Evol. 2016 Jun 11;8(7):2076–92. doi: 10.1093/gbe/evw132 (PMC4987104; doi:10.1093/gbe/evw132)
Supplement: Supplementary Data [file supp_8_7_2076__index.html]

Emergence and Evolution of Hominidae-Specific Coding and Noncoding Genomic Sequences — Supplementary Data 

# Emergence and Evolution of Hominidae-Specific Coding and Noncoding Genomic Sequences

## Supplementary Data

files

- Supplementary Data - zip file
