# Supplementary material for: Emergence and Evolution of Hominidae-Specific Coding and Noncoding Genomic Sequences
Source: Genome Biol Evol. 2016 Jun 11;8(7):2076–92. doi: 10.1093/gbe/evw132 (PMC4987104; doi:10.1093/gbe/evw132)
Supplement: Supplementary Data [file supp_evw132_suppl_data.zip › Saber_supplementary_material.docx]

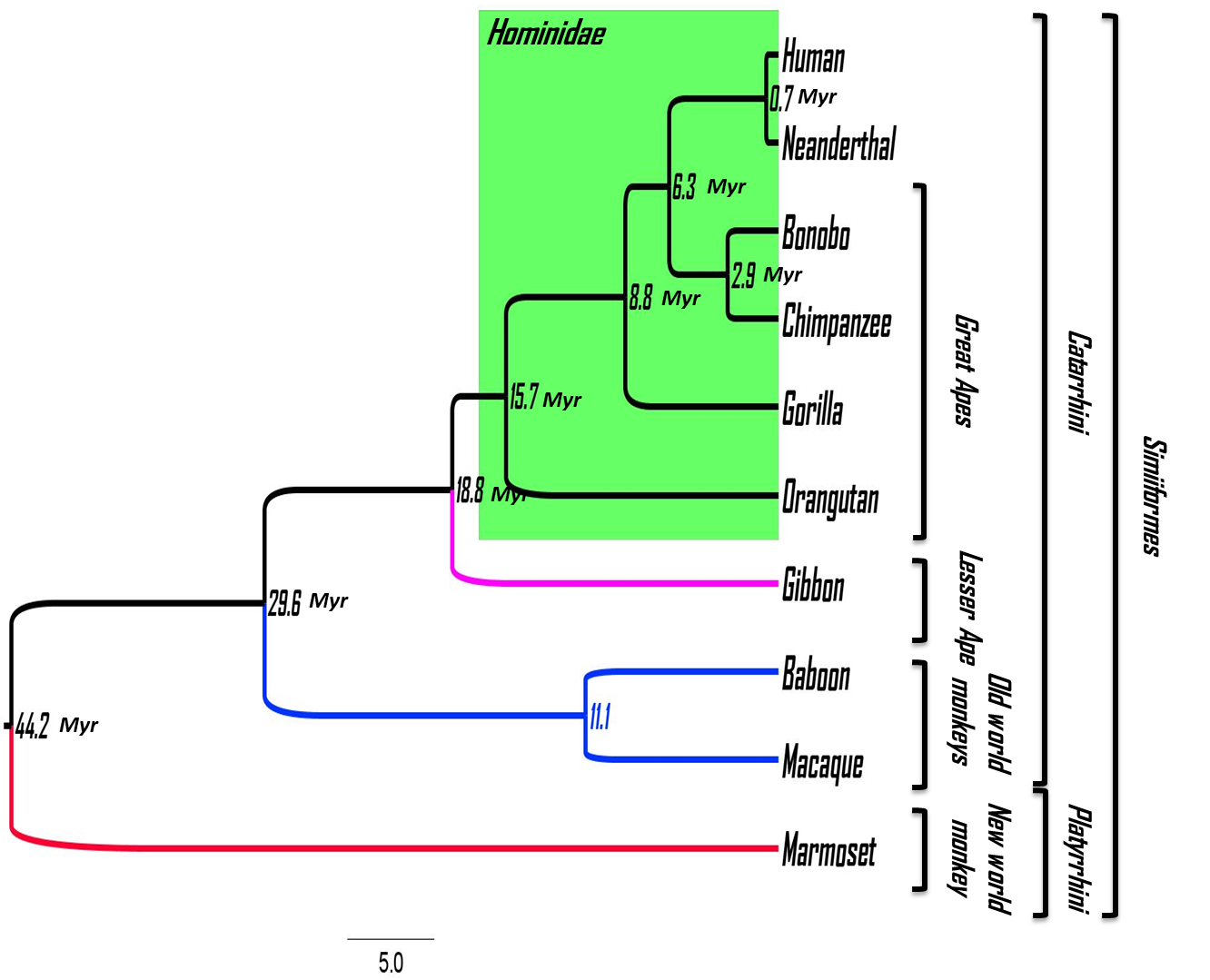


Figure S1 — Molecular phylogeny of simians. Hominidae is one of the two living families of ape superfamily Hominoidea; Hylobatidae or lesser apes constitute the other family. Divergence times (measured as million years ago) were retrieved from Time tree knowledge-base. Pink, blue and red colored branches respectively represent lesser apes (Hylobatidae), old world monkeys (Cercopithecidae) and new world monkeys.

**Table S1.**

**a) Properties of Hominoid-specific genes identified by Xie et al. (2012) over time using Ensembl genomic database.**

| Gene | Ensembl 54  (2009) | Ensembl 75 (2014) | Ensembl 82  (2015) |
| --- | --- | --- | --- |
| ENSG00000174407 | Protein coding§ | Protein coding | Antisense gene |
|  | Cat, dolphin † | gibbon | - |
| ENSG00000204091 | Protein coding | Antisense gene | Antisense gene |
|  | tarsier, mouse lemur, megabat, dolphin, alpaca | - | - |
| ENSG00000212736 | Protein coding | Do not exist | Do not exist |
|  | - |  |  |
| ENSG00000167747 | Protein coding | Protein coding | Protein coding |
|  | Megabat, dolphin | Gibbon, Megabat , dolphin | Gibbon, Megabat, Vervet-agm, dolphin |
| ENSG00000214112 | Protein coding | Do not exist | Do not exist |
|  | hyrax, megabat, tarsier, dolphin, elephant, cat |  |  |
| ENSG00000221891 | Protein coding | Processed Pseudogene | Processed Pseudogene |
|  | - |  |  |

**§**  ***Gene type.***

† ***Species other than Hominidae in which significant conservation and coding potential identified***

**b) Properties of Hominidae-specific genes identified in current study over time using Ensembl genomic database.**

| Gene | Ensembl 54  (2009) | Ensembl 75  (2014) | Ensembl 82  (2015) |
| --- | --- | --- | --- |
| ***ENSG00000184029*** | ***Protein coding***§ | ***Protein coding*** | ***Protein coding*** |
|  | ***-***† | ***-*** | ***-*** |

**§**  ***Gene type.***

† ***Species other than Hominidae in which significant conservation and coding potential identified***

**Table S2**. **Non Hominidae species considered for identification of Hominidae-specific genes**

| *Species* |  | | *Build* | | *Species* |  | *Build* |
| --- | --- | --- | --- | --- | --- | --- | --- |
| Alpaca | | Vicugna pacos | | vicPac1 | **Marmoset** | Callithrix jacchus | C_jacchus3.2.1 |
| Anole Lizard | | Anolis carolinensis | | AnoCar2.0 | **Medaka** | Oryzias latipes | MEDAKA1 |
| Armadillo | | Dasypus novemcinctus | | Dasnov3.0 | **Megabat** | Pteropus vampyrus | pteVam1 |
| Atlantic Cod | | Gadus morhua | | gadMor1 | **Microbat** | Myotis lucifugus | Myoluc2.0 |
| Bushbaby | | Otolemur garnettii | | OtoGar3 | **Mouse** | Mus musculus | GRCm38 |
| Caenorhabditis elegans | | Caenorhabditis elegans | | WBcel235 | **Mouse Lemur** | Microcebus murinus | micMur1 |
| Cat | | Felis catus | | Felis_Catus_6.2 | **Nile tilapia** | Oreochromis niloticus | Orenil1.0 |
| Cave fish | | Astyanax mexicanus | | AstMex102 | **Opossum** | Monodelphis domestica | BROADO5 |
| Chicken | | Gallus gallus | | Galgal4 | **Panda** | Ailuropoda melanoleuca | ailMel1 |
| Chinese softshell turtle | | Pelodiscus sinensis | | PelSin_1.0 | **Pig** | Sus scrofa | Sscrofa10.2 |
| Ciona intestinalis | | Ciona intestinalis | | KH | **Pika** | Ochotona princeps | pika |
| Ciona savignyi | | Ciona savignyi | | CSAV2.0 | **Platyfish** | Xiphophorus maculatus | Xipmac4.4.2 |
| Coelacanth | | Latimeria chalumnae | | LatCha1 | **Platypus** | Ornithorhynchus anatinus | OANA5 |
| Common Shrew | | Sorex araneus | | COMMON_SHREW1 | **Rabbit** | Oryctolagus cuniculus | OryCun2.0 |
| Cow | | Bos taurus | | UMD3.1 | **Rat** | Rattus norvegicus | Rnor_5.0 |
| Dog | | Canis lupus familiaris | | CanFam3.1 | **Rock Hyrax** | Procavia capensis | proCap1 |
| Dolphin | | Tursiops truncatus | | turTur1 | **Sheep** | Ovis aries | Oar_v3.1 |
| Duck | | Anas platyrhynchos | | BGI_Duck_1.0 | **Sloth** | Choloepus hoffmanni | choHof1 |
| Drosophila | | Drosophila melanogaster | | BDGP5 | **Spotted gar** | Lepisosteus oculatus | LepOcu1 |
| Elephant | | Loxodonta africana | | loxAfr3 | **Squirrel** | Ictidomys tridecemlineatus | Spetri2 |
| Ferret | | Mustela putorius furo | | MusPutFur1.0 | **Stickleback** | Gasterosteus aculeatus | BROADS1 |
| Flycatcher | | Ficedula albicollis | | FicAlb1.4 | **Tarsier** | Tarsius syrichta | tarSyr1 |
| Fugu | | Takifugu rubripes | | FUGU4 | **Tasmanian Devil** | Sarcophilus harrisii | DEVIL7.0 |
| Gibbon | | Nomascus leucogenys | | NLeu1.0 | **Tetraodon** | Tetraodon nigroviridis | TETRAODON8 |
| Guinea Pig | | Cavia porcellus | | CavPor3 | **Tree Shrew** | Tupaia belangeri | TREESHREW |
| Hedgehog | | Erinaceus europaeus | | HEDGEHOG | **Turkey** | Meleagris gallopavo | UMD2 |
| Horse | | Equus caballus | | EquCab2 | **Wallaby** | Macropus eugenii | Meug_1.0 |
| Kangaroo Rat | | Dipodomys ordii | | dipOrd1 | **Xenopus** | Xenopus tropicalis | JGI_4.2 |
| Lamprey | | Petromyzon marinus | | Pmarinus_7.0 | **Yeast** | Saccharomyces cerevisiae | R64_1-1 |
| Lesser hedgehog tenrec | | Echinops telfairi | | TENREC | **Zebra Finch** | Taeniopygia guttata | taeGut_3.2.4 |
| Macaque | | Macaca mulatta | | MMUL_1 | **Zebrafish** | Danio rerio | Zv9 |


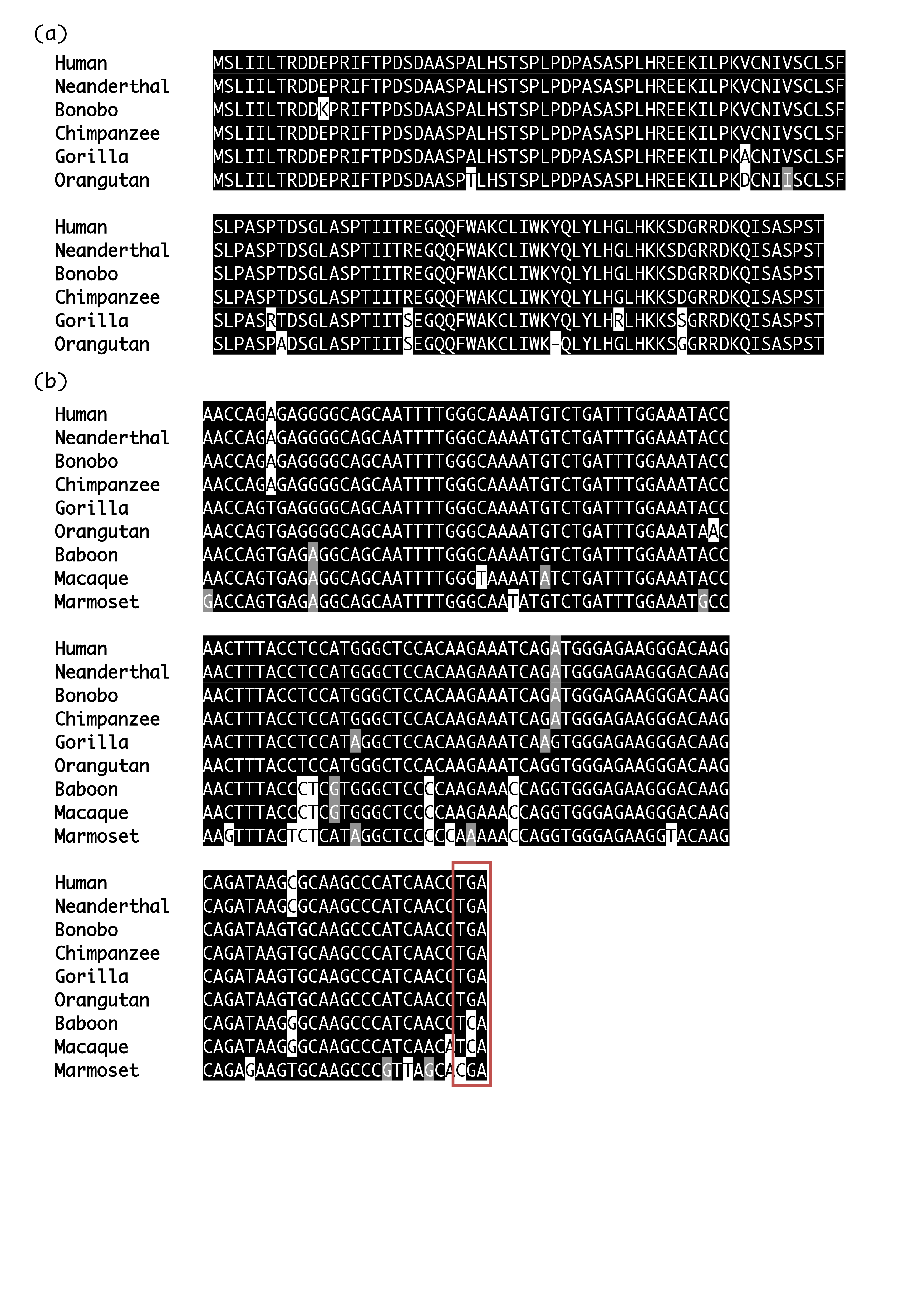


**Figure S2 — DSCR4 sequence alignment.** (a) DSCR4-coded protein’s multiple sequence alignment. (b) Multiple sequence alignment of homologous DNA sequences to human DSCR4 exon 3 protein coding sequence. Common disabler is marked in red rectangle.


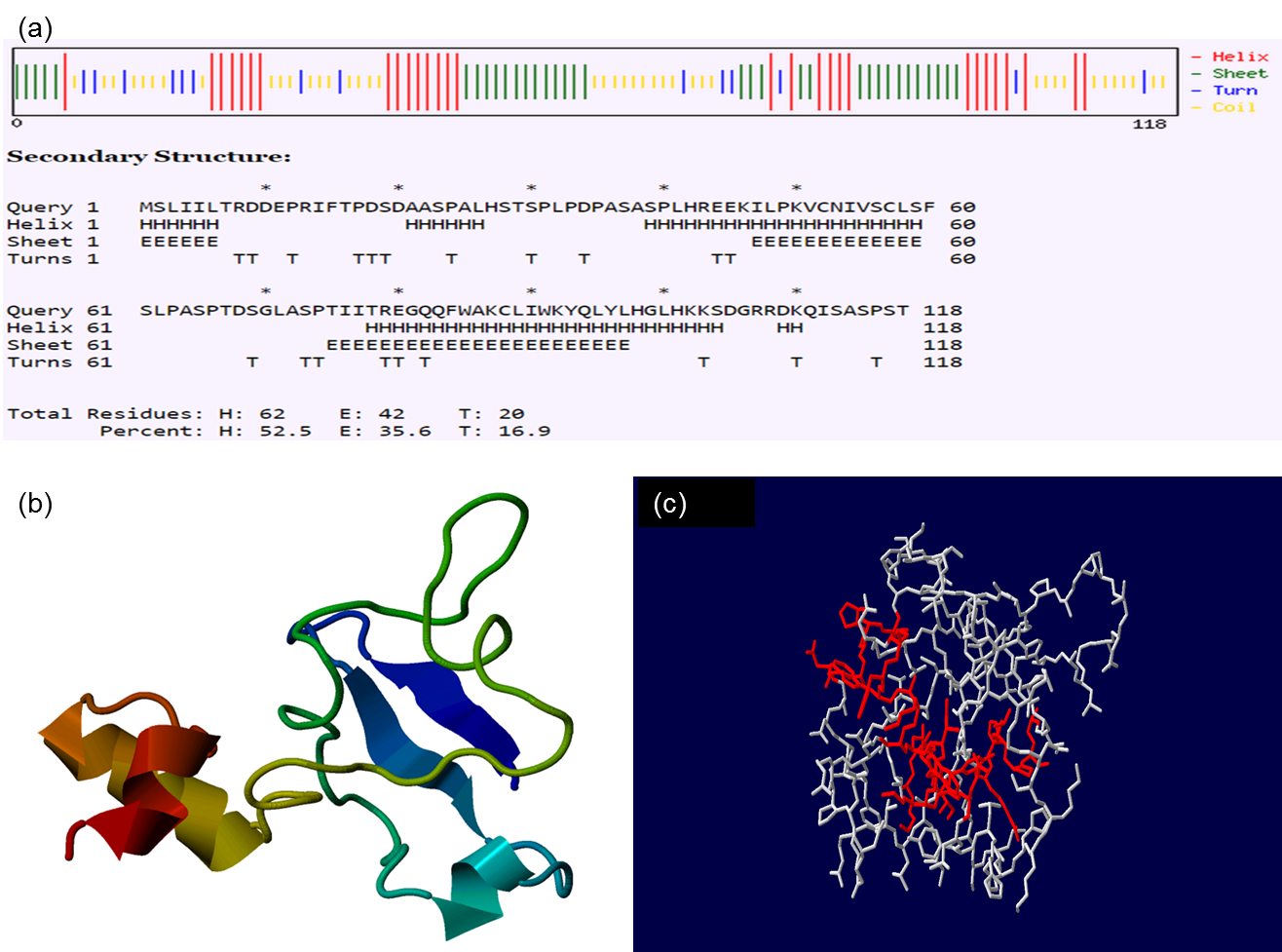


**Figure S3**. (a) Secondary structure analysis of DSCR4 protein using chou and Fasman secondary structure prediction algorithm. (b) DSCR4 secondary structure prediction using 2kjda protein as template. (c) DSCR4 secondary structure prediction using I-TASSER

**TableS3.**  Contribution of transposable elements in HS genes’ exon formation

|  | **Gene coordinate** | | | **TE match in human genome** | | | **TE properties** | |
| --- | --- | --- | --- | --- | --- | --- | --- | --- |
| **Genomic region** | **Chromosome** | **Start** | **Length** | **SW SCORE** | **Start** | **End** | **Class** | **Family** |
| DSCR4/ DSCR8 promoter | 21 | 39493455 | 92 | 2102 | 39493281 | 39494097 | LTR9 | LTR/ERV1 |
| DSCR4 EXON1 | 21 | 39493222 | 233 | 2102 | 39493281 | 39494097 | LTR9 | LTR/ERV1 |
| DSCR4 EXON1 | 21 | 39493222 | 233 | 940 | 39493141 | 39493280 | LTR16A | LTR/ERVL |
| DSCR4 EXON2 | 21 | 39492401 | 102 | 1849 | 39492309 | 39492672 | MLT2C1 | LTR/ERVL |
| DSCR4 EXON3 | 21 | 39426313 | 763 | 460 | 39426799 | 39426943 | LTR79 | LTR/ERVL |


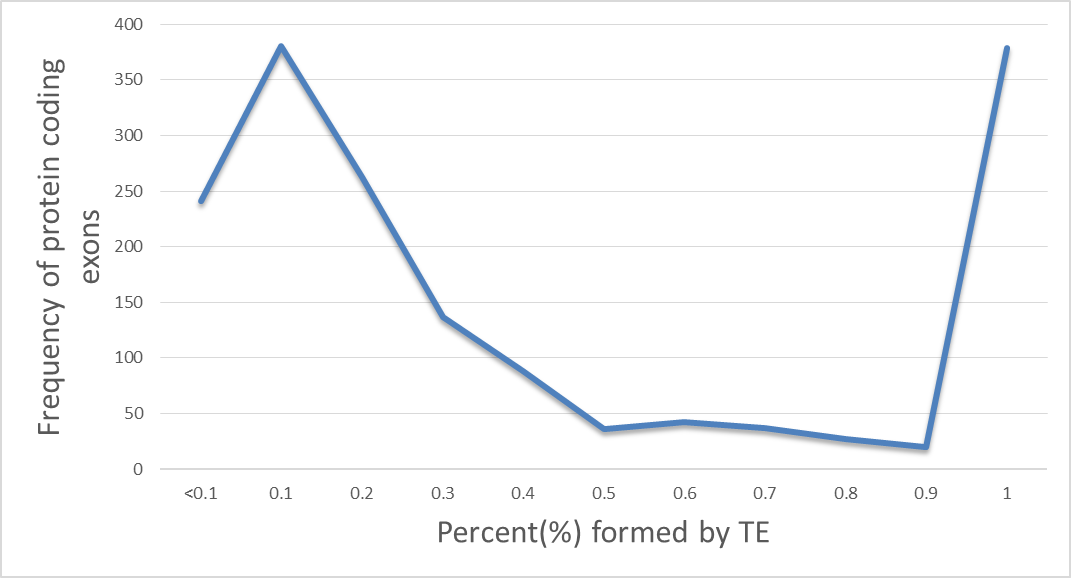


**Figure S4**. **Contribution of transposable elements to human protein coding genes’ exon formation.**


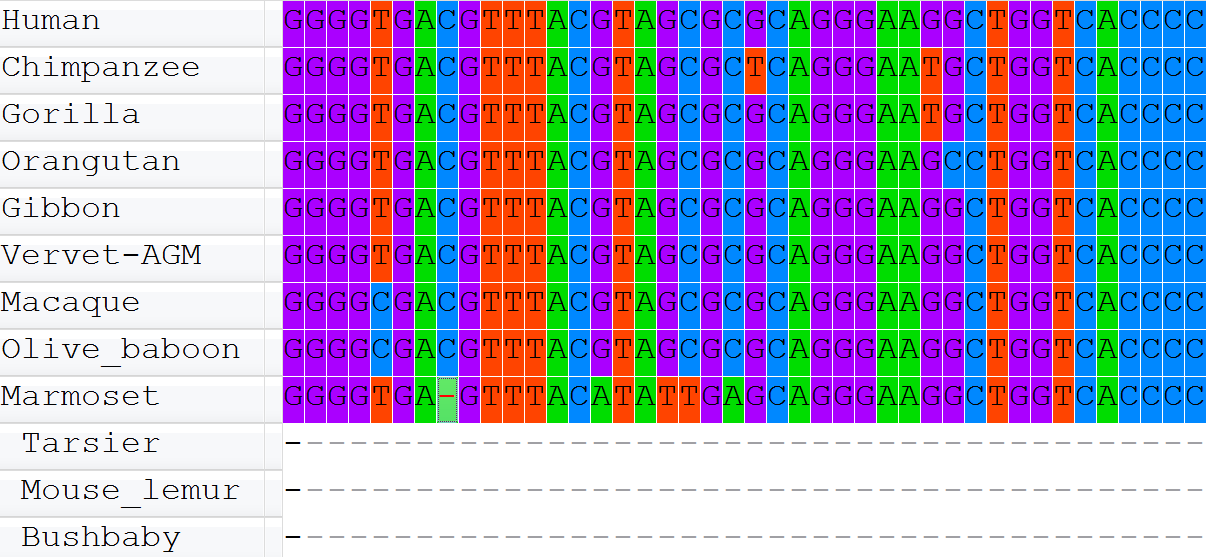


**Figure S5: Multiple alignment of DSCR4 core promoter.** Analysis of the core promoteric region of DSCR4/8 bidirectional promoter reveals that DSCR4 promoteric region has retrotransposed 29-43 million years ago in common ancestor of primates.


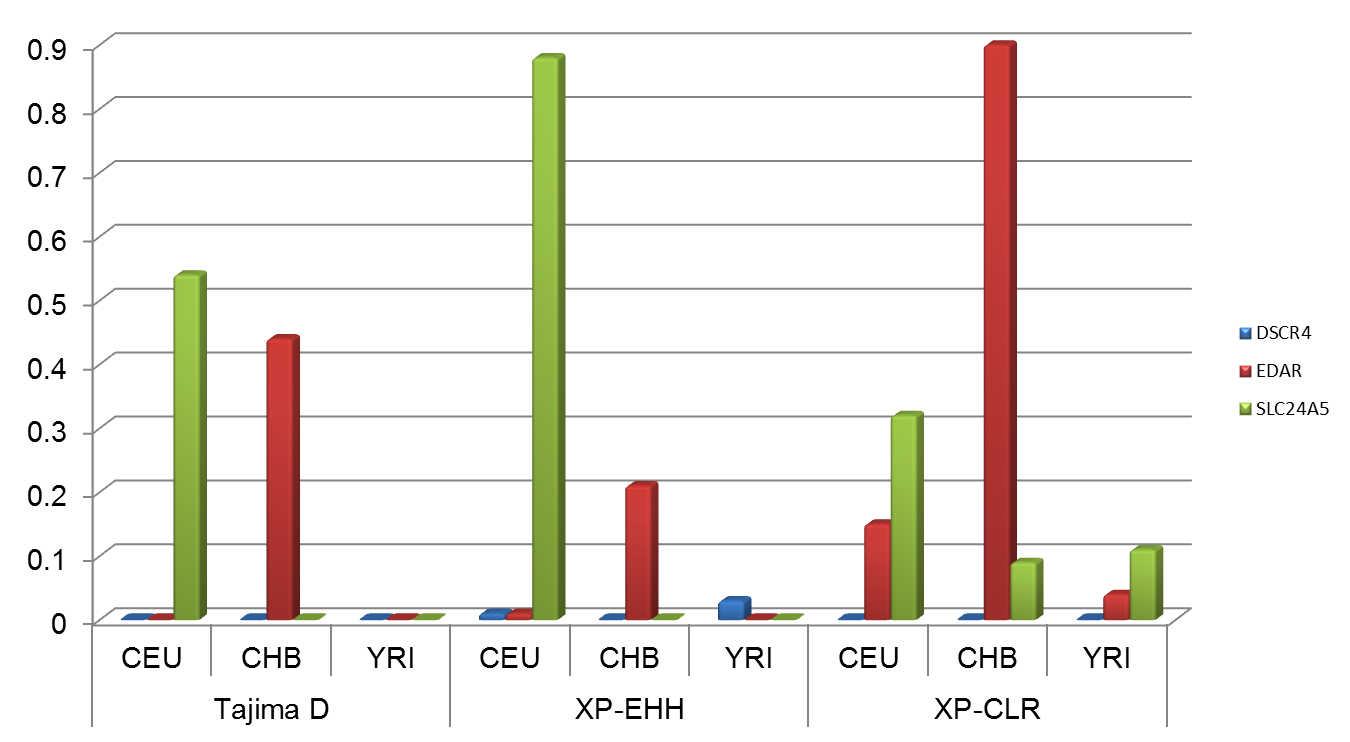


**Figure S6**. **DSCR4 Analysis of selection based on population genomic variation data**. The percentage of windows under positive selection based on Tajima D and XP-CLR as well as percentage of SNPs under positive selection based on XP-EHH in European, Asian and African populations for HS gene, DSCR4 along with SLC24A5 and EDAR genes which have been shown to be under positive selection respectively in European and Asian populations, are shown as bar chart. (CEU: Utah residents with Northern and Western European ancestry from the CEPH collection, CHB: Han Chinese in Beijing, China, YRI: Yoruba in Ibadan, Nigeria). DSCR4 doesn’t show signs of positive selection in any of the investigated populations.


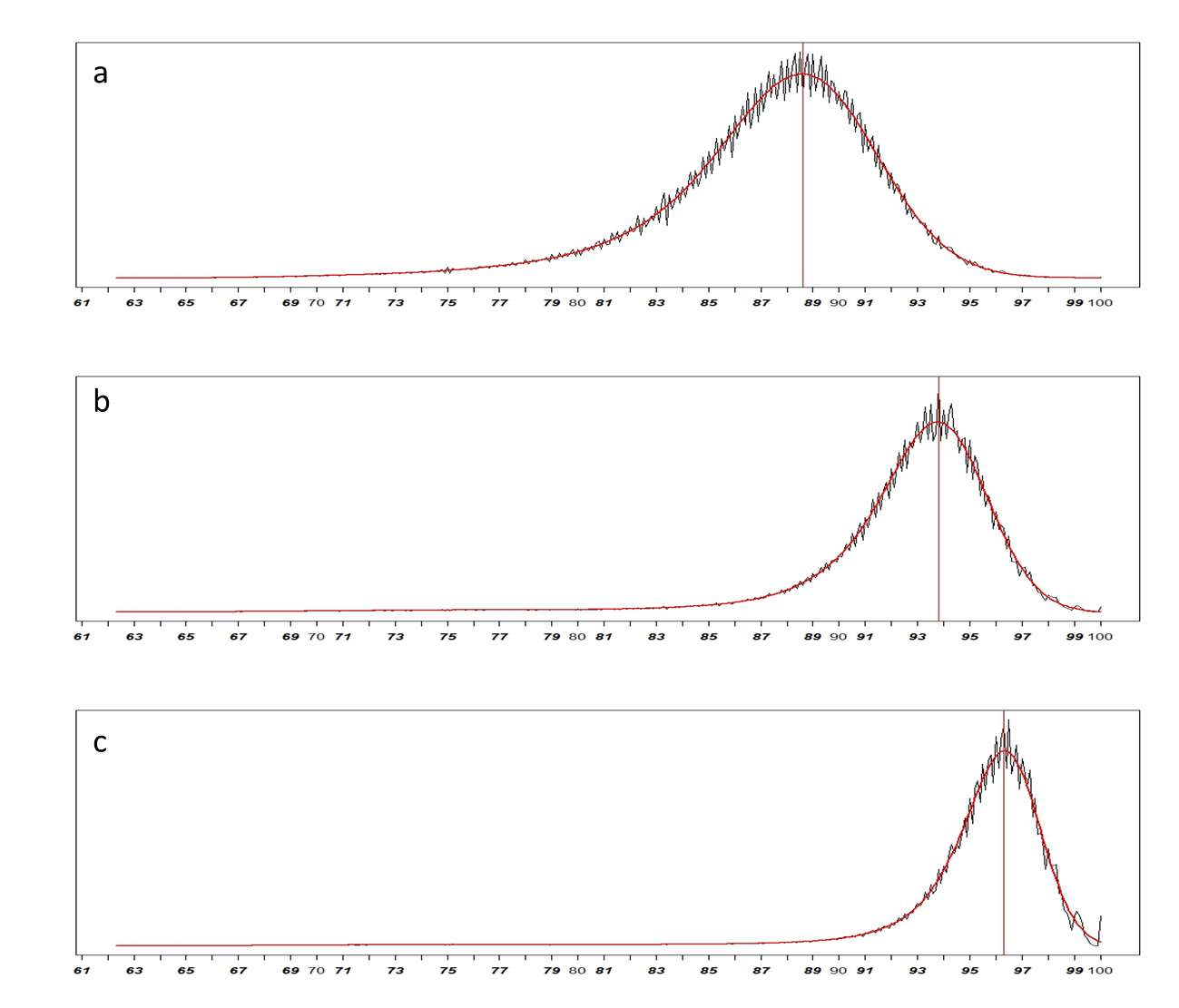


**Figure S7**. **Neutrally evolving sequences’ conservation level in Non-coding sequences**. Human-marmoset, human-macaque and human-gibbon homologous sequences’ conservation plot based on non-coding DNA conservation level (a, b and c, respectively).

A


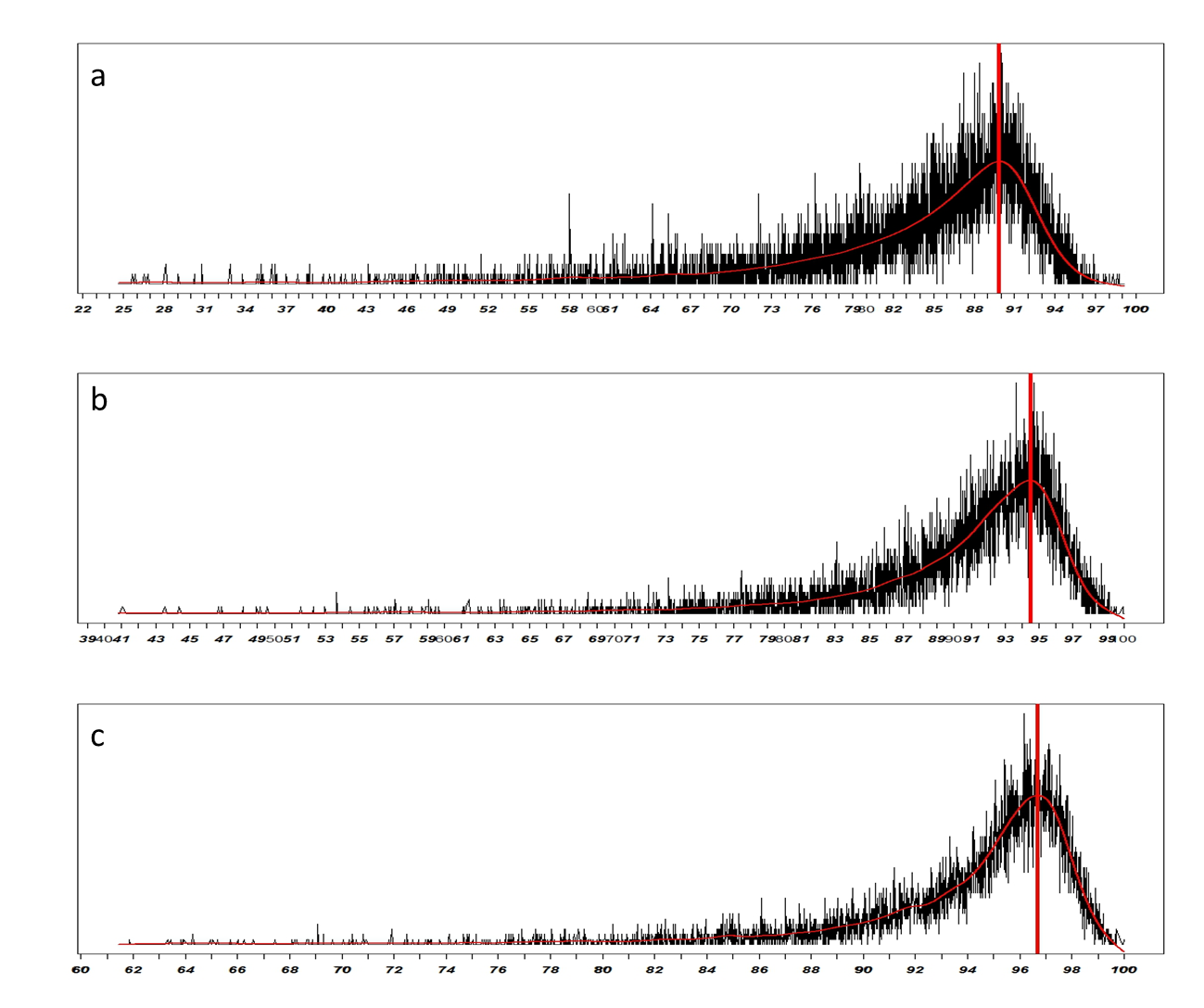


**Figure S8**. **Neutrally evolving sequences’ conservation level in coding sequences**. Human-marmoset, human-macaque and human-gibbon homologous sequences’ conservation plot based on protein coding sequences synonymous sites substitution rate (a, b and c respectively)

A


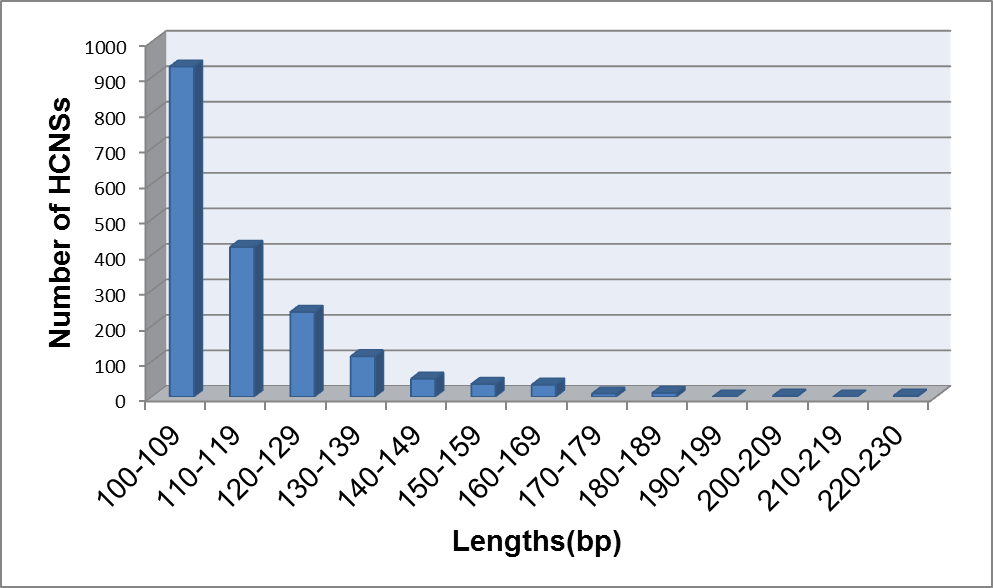


**Figure S9**. **Length distribution of HS HCNSs**.


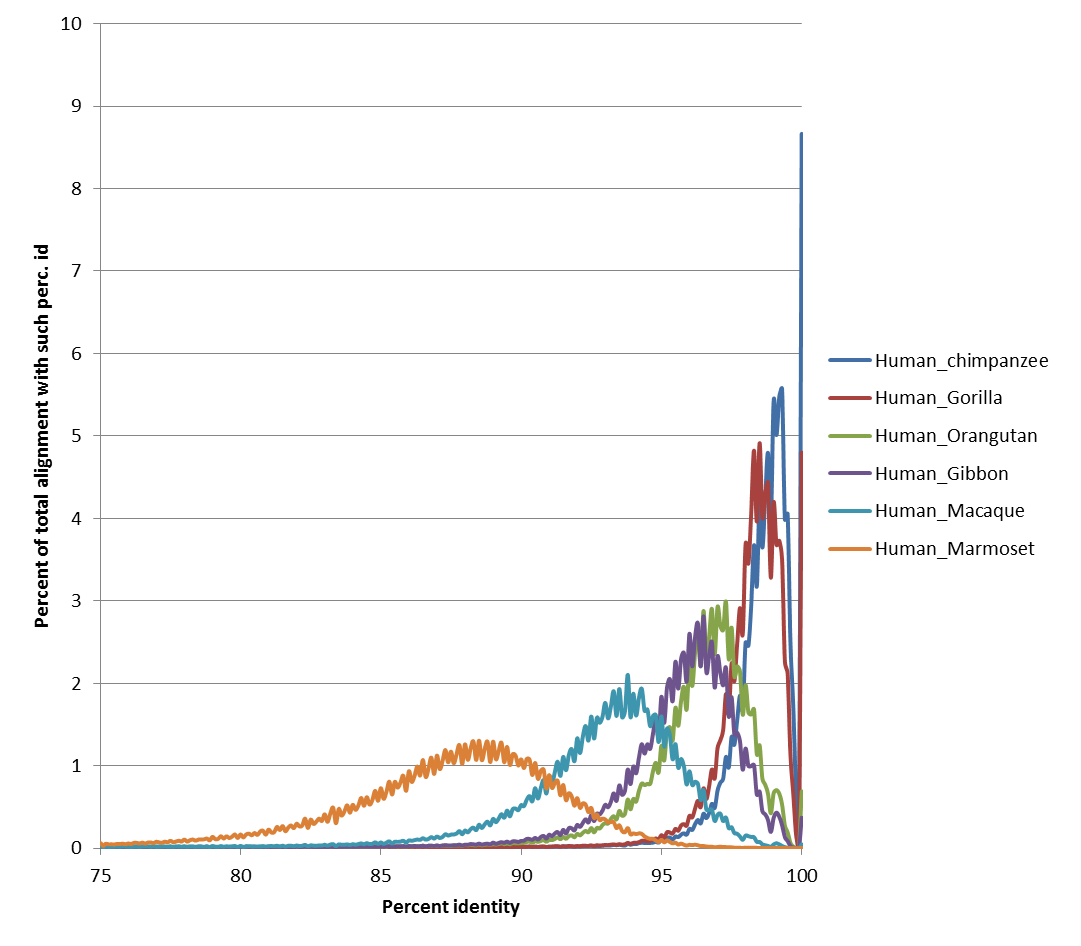


**Figure S10. Noncoding identity distribution within Catarrhini infra order.** Distributions of whole-genome noncoding sequence identities are represented for Catarahini members.


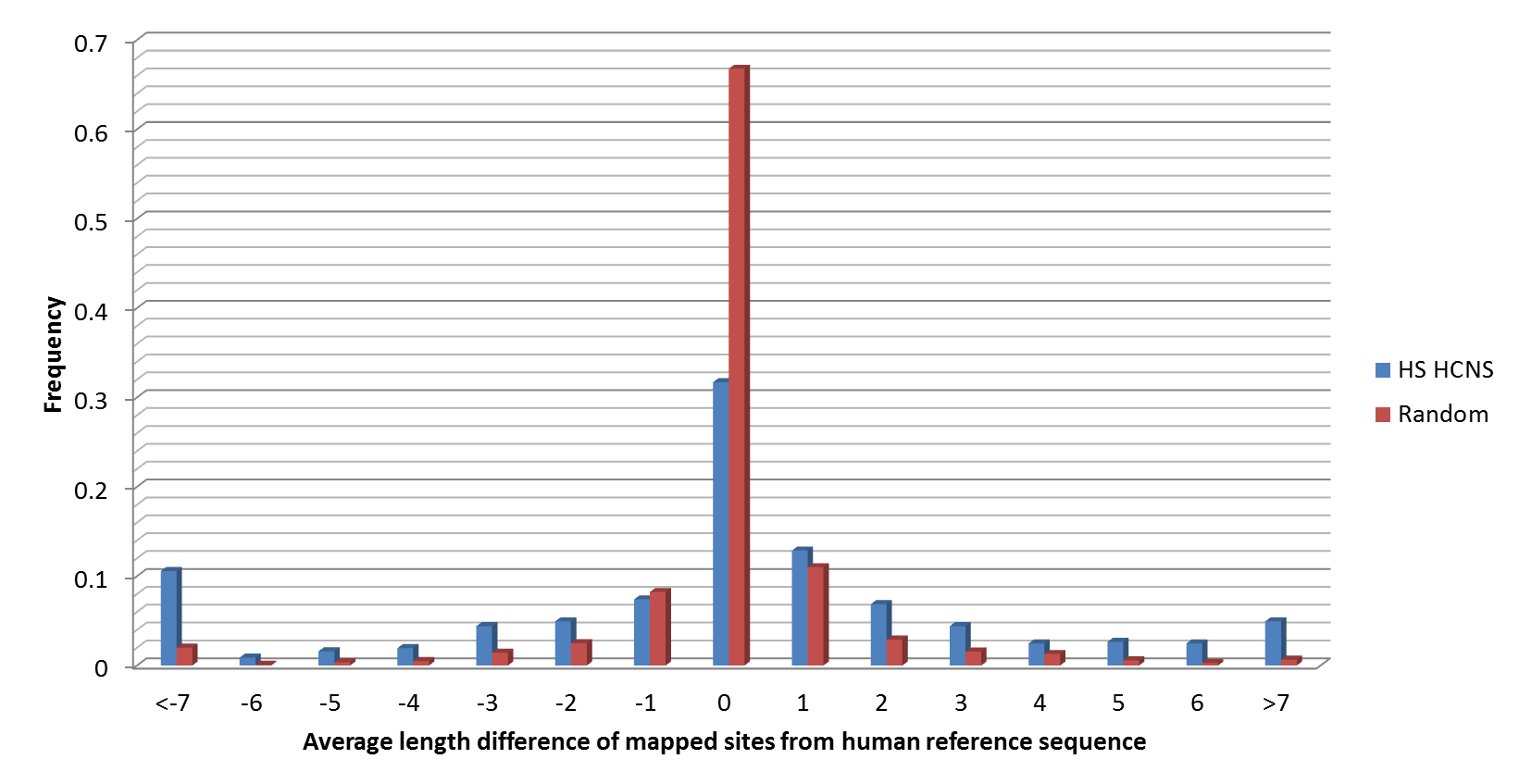


**Figure S11**. **Evolutionary origin of HS HCNSs**. Average length difference of sequences mapped to HS HCNSs in gibbon and rhesus macaque from human reference sequence is significantly higher than random sequences of the same number and size.

Table S4. Examples of HS HCNSs under strong accelerated evolution in Hominidae common ancestor

| HS HCNS properties | | | Genetic distance | Likely target gene | | |
| --- | --- | --- | --- | --- | --- | --- |
| Chromosome | Start | Length | α distance | Gene name | Start | End |
| 3 | 43045007 | 107 | 0.19 | KRBOX1 | 42850938 | 43097363 |
| X | 152417953 | 165 | 0.12 | MAGEA1 | 152481522 | 152486115 |
| 11 | 49108620 | 100 | 0.11 | TRIM64C | 49075266 | 49080664 |
| 2 | 131062944 | 105 | 0.1 | CCDC115 | 131095814 | 131099922 |
| X | 119157282 | 110 | 0.1 | RHOXF2B | 119205848 | 119211707 |
| 1 | 13214501 | 116 | 0.1 | PRAMEF26 | 13216356 | 13219581 |
| 16 | 33769118 | 101 | 0.09 | RP11-812E19.9 | 33647044 | 33647696 |
| 16 | 33744583 | 100 | 0.09 | RP11-812E19.9 | 33647044 | 33647696 |
| 16 | 32922979 | 100 | 0.09 | TP53TG3 | 32684852 | 32688053 |
| 3 | 14132416 | 112 | 0.08 | TPRXL | 13978756 | 14124311 |
| 7 | 62537666 | 109 | 0.08 | AC006455.1 | 62809239 | 62812151 |
| 5 | 70988362 | 107 | 0.08 | CARTPT | 71014990 | 71016875 |
| 11 | 104943827 | 124 | 0.08 | CASP1 | 104896170 | 104972158 |
| 14 | 74764127 | 133 | 0.07 | ABCD4 | 74752126 | 74769759 |
| 18 | 76749706 | 105 | 0.07 | SALL3 | 76740275 | 76762677 |
| 7 | 62778744 | 123 | 0.06 | AC006455.1 | 62809239 | 62812151 |
| 10 | 27618391 | 120 | 0.06 | PTCHD3 | 27687116 | 27703297 |
| 2 | 132093021 | 108 | 0.06 | PLEKHB2 | 131862420 | 132111282 |
| 7 | 48121826 | 120 | 0.06 | UPP1 | 48128225 | 48148330 |
| 17 | 26048502 | 109 | 0.06 | NOS2 | 26083792 | 26127525 |
| 8 | 21426860 | 107 | 0.06 | GFRA2 | 21547915 | 21669869 |
| 2 | 132106021 | 135 | 0.06 | PLEKHB2 | 131862420 | 132111282 |
| 6 | 155929553 | 107 | 0.06 | NOX3 | 155716504 | 155777037 |
| 7 | 63149048 | 112 | 0.06 | AC073188.1 | 62858414 | 62858860 |

**Figure S12: The GC content of the CNS and CNS flanking regions of CNSs.** Using sliding windows of 200bp size and sliding steps of 10bp, the percent GC contents of the HS HCNS a and flanking regions were computed. Position 0 is the 100bp in the center of the CNSs.

**a**


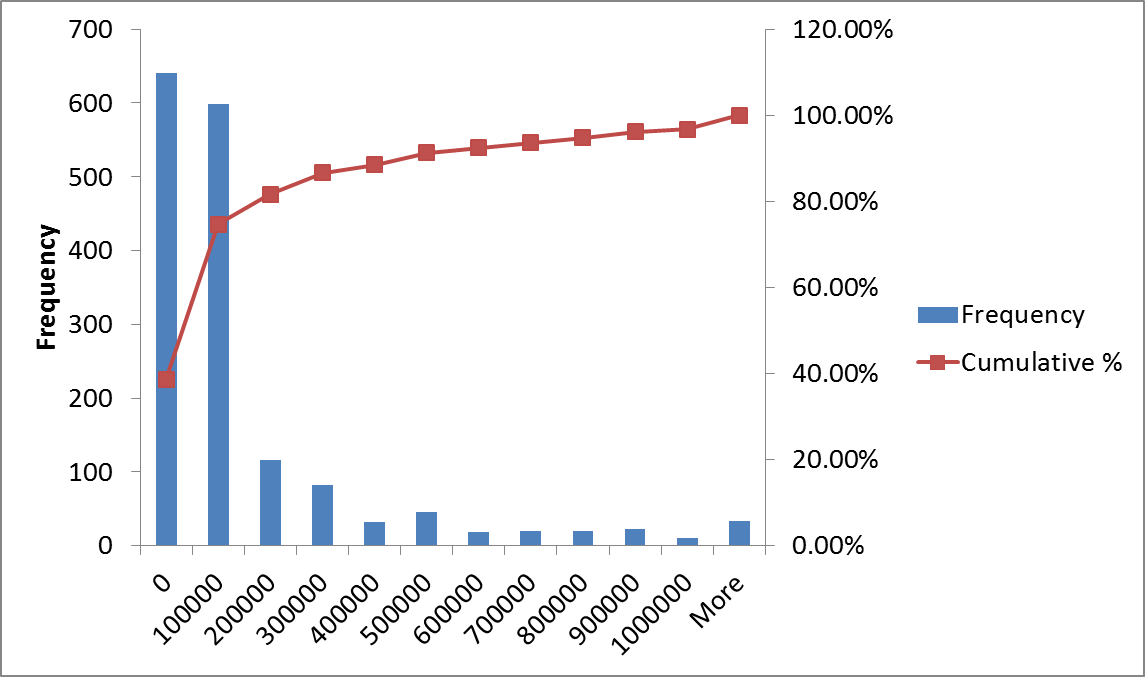


**b**


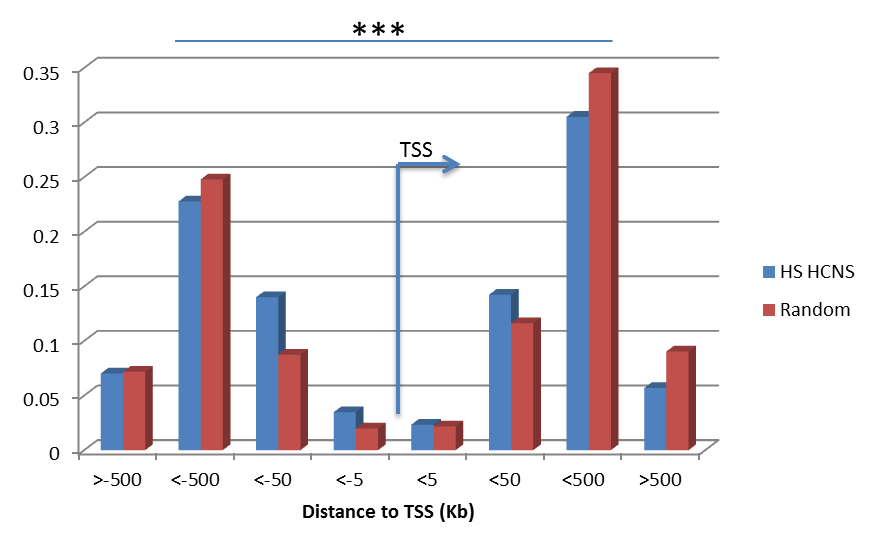


**Figure S13**. (a) The proximity of the HS HCNSs to genes. The horizontal axis represents the distance of the CNS to the closest protein coding gene. (b) The proximity of the HS HCNSs to Transcription start site (TSS) compared to random expectations using GREAT online software. HS HCNSs are significantly overrepresented within range of <50 Kb from TSSs, and underrepresented for distance ranges > 50 kb from TSSs (p-value < 2.2e-16, chi-square test).

Table S5. HS HCNSs target genes with expression switch on the branch connecting great apes and macaque.

| Organ | Gene ID | HS HCNS per target gene |
| --- | --- | --- |
| Brain | ENSG00000183098, ENSG00000179163, ENSG00000105675 | 1 |
| Cerebellum | ENSG00000183098, ENSG00000112679, ENSG00000169851, ENSG00000169851, ENSG00000086827, ENSG00000146938, ENSG00000169504, ENSG00000169504, ENSG00000124198, ENSG00000165194, ENSG00000165300, ENSG00000165300, ENSG00000213949, ENSG00000162415, ENSG00000083123, ENSG00000105675, ENSG00000184220, ENSG00000146085, ENSG00000146085, ENSG00000111261, ENSG00000114480, ENSG00000114480, ENSG00000060718, ENSG00000143061, ENSG00000143061, ENSG00000174989, ENSG00000165194, ENSG00000165194, ENSG00000165194, ENSG00000204262, ENSG00000196950, ENSG00000166897, ENSG00000206052, ENSG00000166266, ENSG00000169851, ENSG00000087502, ENSG00000133687, ENSG00000169851, ENSG00000108018 | 2.6 |
| Heart | ENSG00000149418 | 1 |
| Liver | ENSG00000183098, ENSG00000112139, ENSG00000131142, ENSG00000144028, ENSG00000140479, ENSG00000139263, ENSG00000197766, ENSG00000172348, ENSG00000166377, ENSG00000077380, ENSG00000122641, ENSG00000122641, ENSG00000106070, ENSG00000113273, ENSG00000110900, ENSG00000110900, ENSG00000171714 | 1.4 |
| Testis | ENSG00000183098, ENSG00000184226, ENSG00000184226, ENSG00000153822, ENSG00000086827, ENSG00000179163, ENSG00000124198, ENSG00000112996, ENSG00000215009, ENSG00000186094, ENSG00000163528, ENSG00000141622, ENSG00000135541, ENSG00000185008, ENSG00000198597, ENSG00000138316, ENSG00000085382, ENSG00000170417, ENSG00000071242, ENSG00000071242, ENSG00000166377, ENSG00000197603, ENSG00000157330, ENSG00000187391, ENSG00000145439, ENSG00000174429, ENSG00000125999, ENSG00000143222, ENSG00000169946, ENSG00000143507, ENSG00000065325, ENSG00000141568, ENSG00000188517, ENSG00000173200, ENSG00000147202, ENSG00000204262, ENSG00000106070, ENSG00000132842, ENSG00000164326, ENSG00000180537, ENSG00000087502, ENSG00000184178, ENSG00000185008, ENSG00000163995, ENSG00000038532, ENSG00000152936, ENSG00000152936, ENSG00000152936 | 1.4 |


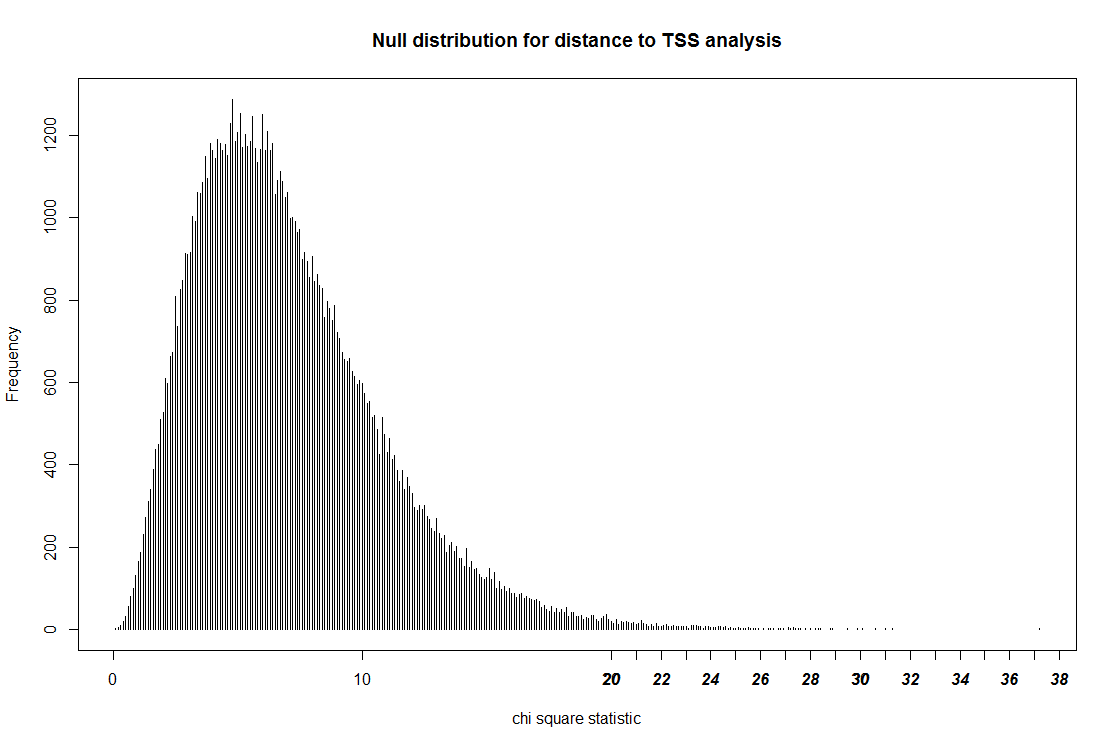


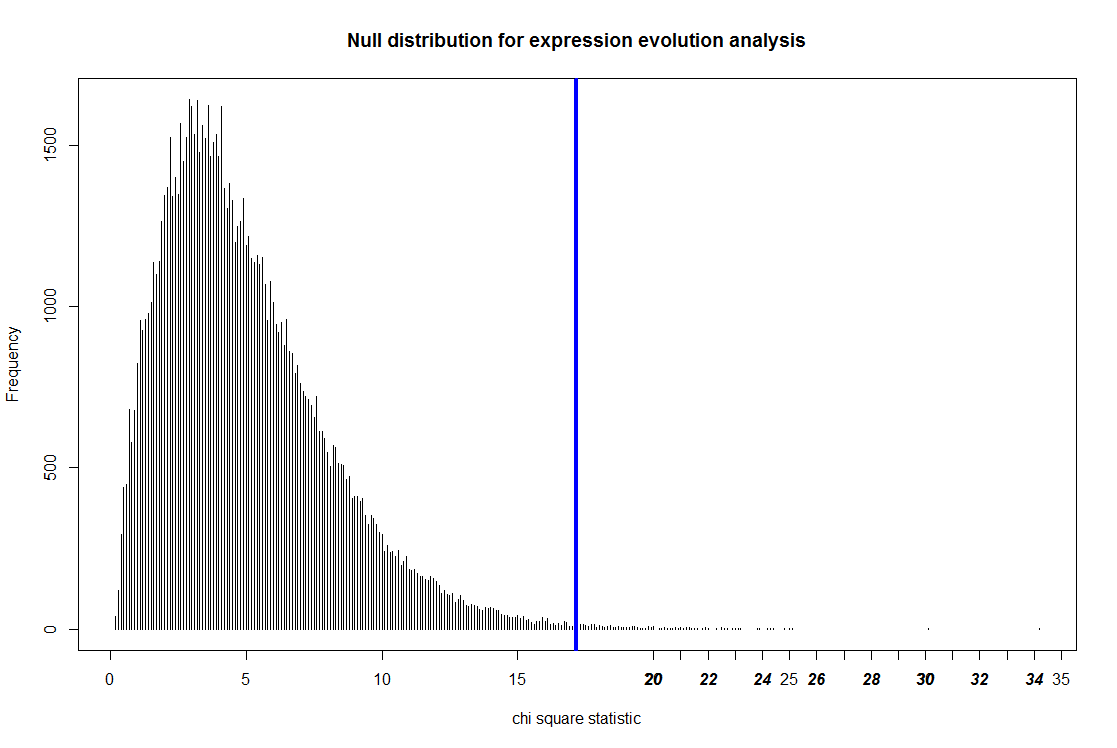


**Figure S14**. (**a)** Monte Carlo simulated chi square test null distribution for distance of HS HCNSs to closest Transcription Start Site (TSS). Observed test statistic was calculated as 183.62 (not shown). Empirical p-value calculated based on 1e+05 replicates, is equal to 1e-05. (**b)** Monte Carlo simulated chi square test null distribution for expression evolution analysis. Observed test statistic was calculated as 17.139 (blue vertical line). Empirical p-value calculated based on 1e+05 replicates, is equal to 0.00767.


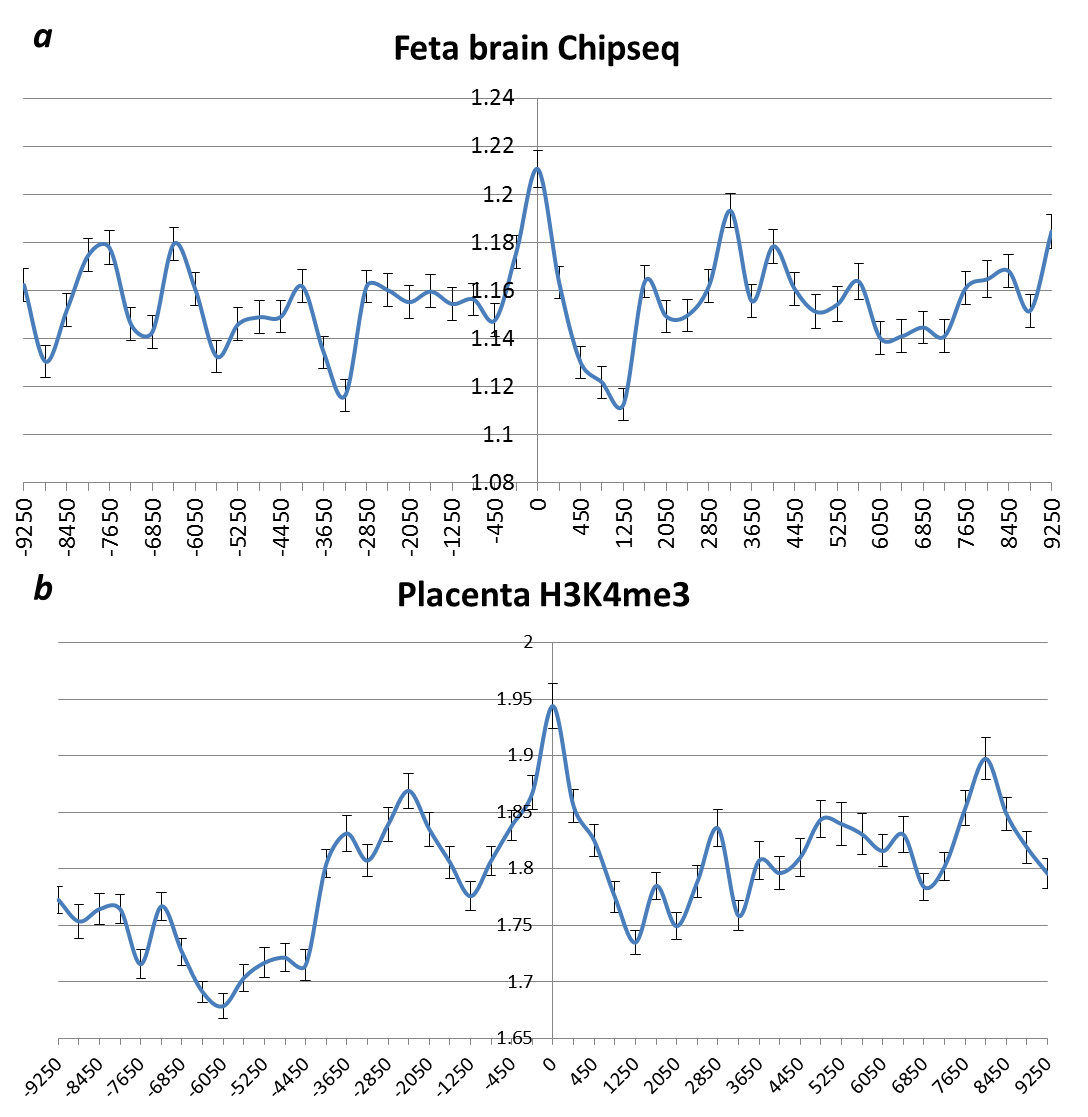


**Figure S15. Analysis of tissue specificity of HS HCNSs. (a)** Analysis of the Epigenome roadmap chromatin immunoprecipitation data reveals intensified chipseq signal within HS HCNSs compared to flanking regions in fetal brain. (b) Analysis of the Epigenome roadmap data regarding H3K4me3 epigenetic mark showed intensified signal within HS HCNS in placenta. H3K4me3 is associated with active promoter regions.

**Table S6.**

(a) Gene ontology analysis of conserved noncoding sequences under accelerated evolution in Human (HACNs).

| Biological Process | Binom Fold Enrichment |
| --- | --- |
| regulation of type B pancreatic cell development | 6.67 |
| negative regulation of transcription by competitive promoter binding | 4.99 |
| forebrain ventricular zone progenitor cell division | 4.64 |
| regulation of transcription involved in cell fate commitment | 3.48 |
| positive regulation of filopodium assembly | 3.35 |
| neuron recognition | 3.13 |
| neuron fate specification | 2.84 |
| regulation of filopodium assembly | 2.68 |
| neural crest cell migration | 2.39 |
| forebrain neuron differentiation | 2.32 |
| cell recognition | 2.3 |
| cell fate specification | 2.2 |
| neural crest cell development | 2.19 |
| negative regulation of neuron differentiation | 2.18 |
| forebrain generation of neurons | 2.18 |
| neuron fate commitment | 2.13 |
| homophilic cell adhesion | 2.08 |

(b) Gene ontology analysis of human genome regions under accelerated evolution (HARs).

| Biological Process | Binom Fold Enrichment |
| --- | --- |
|  |  |
| - | - |


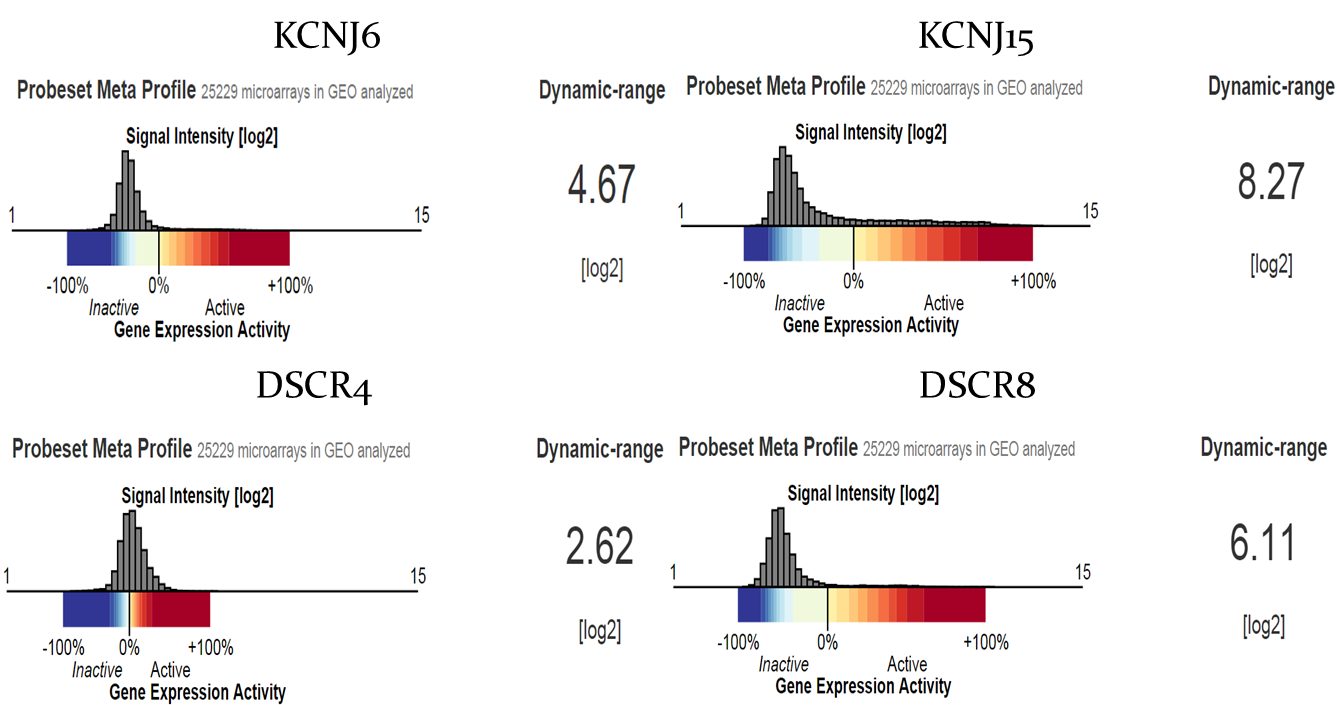


**Figure S16**. **Absolute gene expression profiles of DSCR4 and DSCR8 genes along with their flanking genes based on 25229 Affymetrix Human Genome U133 Plus 2.0 Array analysis.**


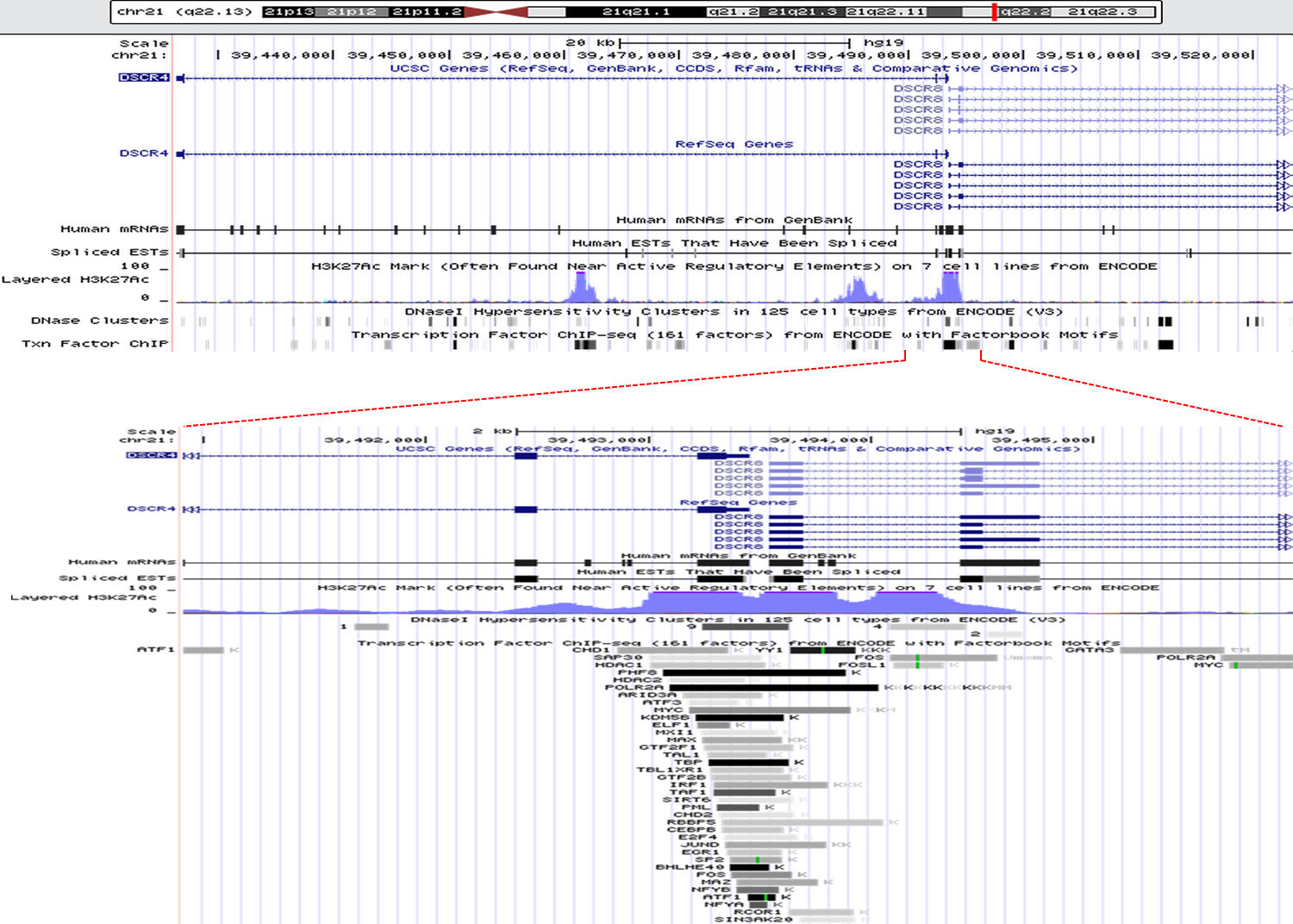


**Figure S17**. **DSCR4 gene regulation**. Encode data suggest existence of three active regulatory elements within DSCR4gene. These regions share three characteristics: h3k27 acylation epigenetic mark that is found near active regulatory elements, forming open chromatin region which is a characteristic shared by several classes of transcription factor binding sites and acting as binding site for several transcription factors.
